# How environmental health burdens shape physical activity patterns: age-specific evidence from OECD countries

**DOI:** 10.1186/s12889-026-27950-9

**Published:** 2026-06-04

**Authors:** Salim Yılmaz, Sumeyra Gundem, Sevde Betul Kara

**Affiliations:** 1 Department of Healthcare Management, Faculty of Health Sciences, Acibadem Mehmet Ali Aydinlar University, Istanbul, 34752 Türkiye; 2 Department of Healthcare Management, Graduate School of Health Sciences, Acibadem Mehmet Ali Aydinlar University, 34752 Istanbul, Türkiye; 3 Department of Healthcare Management, Faculty of Health Sciences, Istanbul Arel University, Istanbul, 34010 Türkiye; 4 Department of Healthcare Management, Institute of Graduate Studies, Istanbul University-Cerrahpaşa, 34320 Istanbul, Türkiye; 5 Department of Physiotherapy and Rehabilitation, Faculty of Health Sciences, Istanbul Arel University, Istanbul, 34010 Türkiye; 6 Department of Physiotherapy and Rehabilitation, Institute of Graduate Studies, Istanbul University-Cerrahpaşa, Istanbul, 34320 Türkiye

**Keywords:** Physical Activities, Environmental Exposure, Air Pollution, Smoking, Age Factors, Health Policy

## Abstract

**Background:**

Despite widespread acknowledgment of physical activity’s (PA) benefits, inactivity remains a significant public health challenge, exacerbated by environmental and socioeconomic factors. This study investigates how environmental health burdens—specifically air pollution and smoking-related disease burdens—alongside socioeconomic conditions shape PA patterns across different life stages in OECD countries.

**Methods:**

Using cross-sectional data from 38 OECD countries, age-stratified beta regression models examined the determinants of PA prevalence among adolescents (ages 11–17), adults (ages 18–69), and older adults (ages 70+). Independent variables included air pollution-related and smoking-related Disability-Adjusted Life Years (DALYs), Body Mass Index (BMI), GDP per capita, urbanization, internet penetration, access to sports facilities, healthcare expenditure, and average annual working hours. Data imputation techniques ensured dataset completeness, supported by auxiliary demographic variables. Robustness checks included multicollinearity assessments, non-linear tests, and sensitivity analyses.

**Results:**

Adolescents exhibited relatively high PA rates, positively influenced by urbanization only when socioeconomic contexts were considered (β=0.119, p=0.031). Adult PA was significantly boosted by greater access to sports facilities (β=0.172, p=0.019), while internet penetration negatively impacted activity levels marginally (β=-0.126, p=0.068). For older adults, PA increased notably with better sports facility access (β=0.178, p=0.001) but declined with higher air pollution-related (β=-0.128, p=0.028) and smoking-related DALYs (β=-0.097, p=0.027), as well as increased internet penetration (β=-0.143, p=0.030).

**Conclusion:**

Determinants of PA vary markedly across age groups and include significant interactions with environmental and socioeconomic factors. Urbanization enhances adolescent PA contingent on supportive environments. In adults and older adults, infrastructure such as sports facilities proves essential, though environmental health burdens significantly limit activity in older populations. Age-specific, environmentally informed public health strategies and infrastructural interventions are critical to sustainably promoting PA across life stages.

**Supplementary Information:**

The online version contains supplementary material available at 10.1186/s12889-026-27950-9.

## Introduction

Physical activity (PA) is any bodily movement that expends energy through skeletal-muscle contractions [1]. Regular PA is associated with broad physical, psychological, social, and cognitive benefits—including lower cardiometabolic risk, improved mental health, and enhanced learning and resilience [2–5]. Conversely, physical inactivity, driven by automation, time scarcity, and car-oriented urban design, has amplified the global burden of non-communicable diseases (NCDs)—including obesity, cardiovascular disease, diabetes, and certain cancers [6–8]. Physical inactivity now contributes directly or indirectly to millions of deaths each year [9] and materially inflates healthcare costs [7].

Despite decades of public-health campaigns, roughly one in four adults and four in five adolescents fail to meet World Health Organization (WHO) guidelines for PA [10–12]. Participation diverges sharply across the life-course: children and adolescents struggle to reach recommended daily intensity levels; adult activity tracks socio-economic gradients; and older adults show the steepest activity decline with advancing age [13–15]. Among young people, PA supports healthy growth and psychological well-being but is hindered by screen time, safety concerns, and rising childhood obesity [2–4, 16–19]. In adults, individual socioeconomic status (SES) and neighborhood infrastructure dominate activity choices [20–22]. In older adults, functional limitations, fear of falling, and limited access to age-appropriate facilities—combined with environmental barriers like air pollution—work together to suppress PA [14, 15, 23].

The literature identifies multifactorial drivers of PA—demographic, psychological, behavioral, socio-cultural, and environmental [24–27]. However, three critical gaps limit the translation of this knowledge into effective policy interventions. First, most cross-national studies treat PA determinants homogeneously across age groups, applying uniform models to populations with fundamentally different activity needs, constraints, and vulnerabilities [22, 25, 28]. This overlooks how the same contextual factor—such as urbanization or internet access—may facilitate PA in one age group while constraining it in another. Second, few studies systematically integrate environmental health burdens into PA models, despite growing evidence that air pollution and smoking-related disease burdens directly limit physical capacity and deter outdoor activity, particularly among vulnerable populations [29–34]. Third, existing research rarely accounts for non-linear relationships between contextual factors and PA outcomes, potentially misspecifying the true nature of these associations and leading to imprecise policy recommendations [35–37]. These gaps have important implications for public health strategy. Without age-stratified evidence, interventions may inadvertently prioritize factors that matter less for specific life stages while neglecting critical determinants. Without incorporating environmental health burdens—quantified through Disability-Adjusted Life Years (DALYs)—policymakers miss opportunities to address the compound effects of poor air quality and tobacco-related morbidity on population activity levels [33, 38, 39]. And without testing for non-linear effects, we risk assuming dose-response relationships where thresholds or saturation points may exist [40, 41].

The present study addresses these gaps through three key contributions. First, we apply age-stratified beta regression models to examine PA prevalence separately among adolescents (11–17 years), adults (18–69 years), and older adults (70 + years) across 38 OECD countries, enabling direct comparison of how shared macro-level factors and age-specific health burdens influence PA across the life-course. Second, we systematically integrate environmental health burden metrics—specifically, air pollution-related and smoking-related DALYs—alongside traditional socioeconomic and built-environment indicators, providing a more comprehensive assessment of the contextual determinants shaping PA patterns. Third, we test for non-linear relationships between key predictors and PA outcomes using polynomial terms and robust diagnostic procedures, revealing potential threshold effects that linear models would miss. By isolating life-stage-specific determinants and examining both linear and non-linear dynamics, this study provides actionable evidence to guide targeted infrastructure investments, environmental regulations, and digital-behavior programs aimed at increasing PA across the lifespan. Our findings demonstrate that effective PA promotion requires moving beyond universal recommendations toward age-tailored, environmentally informed, and context-specific interventions.

## Methods

### Study design

This cross-sectional ecological study analyzes secondary data from 38 OECD countries, stratified by age, to examine how country-level socioeconomic, environmental, and health-system characteristics relate to population-level PA prevalence. The design is suited to research questions that probe macro-contextual determinants and aggregated behavior patterns rather than individual causal pathways. Leveraging cross-national variation, it enables comparisons across the life course and highlights structurally meaningful, policy-relevant factors. While doing so, the approach explicitly acknowledges the inherent limits of ecological analysis—such as the risk of ecological fallacy—while aiming to generate evidence that can inform policy design through cross-country comparisons [42].

### Variable definitions

This study is based on the most recent records and aims to understand the determinants of PA levels across various age groups using a range of explanatory variables. We selected a set of socioeconomic, environmental, and health-related variables that are both theoretically relevant and empirically supported as influential drivers of PA. In our models, the dependent variables are the prevalence rates of PA for three different age groups, operationalized as the proportion of the population meeting the WHO PA recommendations: at least 60 min of moderate-to-vigorous PA daily for adolescents (ages 11–17), and at least 150 min of moderate-intensity activity per week (or equivalent) for adults (ages 18–69) and older adults (ages 70 and above) [43]. It should be noted that while WHO defines adolescents as ages 11–17 for PA prevalence, some environmental health indicators (e.g., air pollution– and smoking-related DALYs) are only available for the 10–19 age group according to the IHME Global Burden of Disease database; therefore, the nearest corresponding age band was used for consistency across datasets. The independent variables examined as explanatory factors include age-specific BMI; air pollution-related DALYs and smoking-related DALYs per 100,000 population obtained from the Institute for Health Metrics and Evaluation (IHME) Global Burden of Disease Study 2021; healthcare expenditure per capita (in current US dollars); access to sports facilities per 100,000 population; average annual working hours; Gross domestic product (GDP) per capita (in current US dollars); urban population share (as a percentage of total population); and internet penetration rate (percentage of the population with internet access) obtained from the International Telecommunication Union (ITU). In addition, three auxiliary variables—the share of the male and female population, and the national NCDs mortality rate—were not included as independent variables in the regression models, but were used exclusively for data preprocessing and imputation procedures. Specifically, these variables supported the generation of demographically weighted and plausible national estimates of age- and sex-specific PA prevalence, especially for countries with incomplete or missing data. Detailed descriptions of all variables and their corresponding secondary data sources are provided in Table 1 [24–28, 43–52]. Comprehensive details on variable selection, conceptual rationale, data imputation procedures, and variables included in each age-stratified regression model are provided in Supplementary Material, Section S1.

**Table 1:** Research variables

Variables	Definition	Time	Source
PA Prevalence (%)	Proportion of population meeting WHO PA guidelines (by age and sex)	2022	WHO
Mortality Rate from NCDs (%)	Mortality rate from NCDs (Cardiovascular, diabetes, cancer etc.)	2022	WHO
GDP per capita (current United Stated Dollar)	Average income per capita (current US$)	2022	World Bank
Prevalence of Obesity among Adults	BMI ≥ 30 (crude estimate) (%)	2022	WHO
Prevalence of Obesity among Children and Adolescents	Prevalence of obesity among children and adolescents, BMI > + 2 standard deviations above the median (crude estimate) (%)	2022	WHO
Urban Population	Urban population (% of total population)	2022	World Bank
Health Spending per capita (current United Stated Dollar)	Current health expenditure per capita (current US$)	2000–2021	World Bank
Hours Worked Annual	Average annual hours actually worked per worker	2010–2022	OECD
Internet penetration rate	Individuals using the Internet (% of population)	2022	ITU
Air Pollution	10–19 years old, 20 + years old, 70 + years old, Global Burden of Disease Study 2021 (GBD 2021) Results	1990–2021	IHME
Smoking	10–19 years old, 20 + years old, 70 + years old, Global Burden of Disease Study 2021 (GBD 2021) Results	1990–2021	IHME
Population, Male (% of total population)	The percentage of the total national population that is male.	2022	World Bank
Population, Female (% of total population)	The percentage of the total national population that is female.	2022	World Bank

*BMI* Body Mass Index, *GDP* Gross Domestic Product, *IHME* Institute for Health Metrics and Evaluation, *ITU* International Telecommunication Union, *NCDs* Non-Communicable Diseases, *OECD* Organisation for Economic Co-operation and Development, *PA* Physical Activity, *WHO* World Health Organization

Given that the explanatory variables (e.g., GDP per capita, urban population %, internet penetration) are measured at the national level, the present analysis adopts an age-stratified ecological approach. This means that age-specific PA prevalence rates are modeled as a function of broader country-level conditions. Such macro-contextual modeling is suitable when individual-level or age-disaggregated covariates are unavailable but cross-country comparisons are still of interest. The aim is not to infer within-country individual-level associations but rather to identify population-level patterns across OECD countries. To support this interpretation, we conducted robustness checks, including cluster-mean centering and intraclass-correlation diagnostics, which indicated that a significant proportion of variation in PA rates is attributable to between-country differences.

GDP per capita is a fundamental socioeconomic determinant of PA, enabling greater investment in sports infrastructure and health promotion [53]. However, economic growth often shifts occupational structures toward sedentary jobs, potentially reducing total activity [54]. Urban population share has dual effects: it increases availability of parks and recreational facilities [55, 56] but also brings traffic congestion and air pollution that may deter outdoor activity [57]. Internet penetration is linked to increased sedentary behavior, particularly among youth [19, 58], though it can facilitate online exercise programs for older adults. Access to sports facilities shows strong positive associations with PA participation across all age groups [59–63]. Age-stratified BMI measures capture the bidirectional relationship between body weight and PA [64, 65]. Air pollution-related DALYs represent both direct barriers to outdoor activity and long-term health consequences [29–34]. Smoking-related DALYs capture cumulative health burdens that reduce PA capacity, especially in older adults [66, 67]. Healthcare expenditure per capita reflects system capacity to support PA through public health campaigns and preventative services [35, 68–70]. Average annual working hours capture time constraints and cultural norms affecting leisure-time PA [20, 21, 71]. Auxiliary variables—female and male population shares, and NCDs mortality rate—were used exclusively during data preprocessing and imputation. Population shares served as weights to produce demographically representative national PA estimates from sex-specific data [43]. NCDs mortality rate improved prediction of missing values in machine learning algorithms (k-NN, MissForest, XGBoost), given its strong epidemiological relationship with PA [11, 72]. This approach, grounded in best practices for imputing missing data [73], ensured analyses were conducted on a maximally complete and representative dataset, enhancing validity and generalizability of cross-national findings.

### Research model

All three age-stratified beta-regression models share a common set of contextual predictors—sports-facility density, GDP per capita, healthcare expenditure, urban-population share, average annual working hours, and internet-penetration rate—while each incorporates age-tailored health indicators. Model 1 (adolescents, 11–17 years) adds childhood-obesity prevalence and adolescent-specific air-pollution DALYs; Model 2 (adults, 18–69 years) substitutes adult BMI and includes adult air-pollution and smoking DALYs; Model 3 (older adults, 70 + years) retains adult BMI but uses 70 + air-pollution and smoking DALYs to capture cumulative exposure in later life. For adolescent models, although PA prevalence follows the WHO definition (ages 11–17), air-pollution and smoking-related DALYs were derived from IHME’s 10–19 age band, the closest available range; this correspondence has been harmonized across datasets for analytical consistency.

For each country $\:i=1,\dots\:,38$ and age group $g \in \{\text{adolescents, adults, older}\},\ y_{ig} \in (0,1)$ is modeled via beta regression with a logit link:

$$y_{ig}\sim\mathrm{Beta}\left(\mu_{ig}\varphi_{g},\,(1-\mu_{ig})\varphi_{g}\right),\quad \mathrm{logit}(\mu_{ig})=\eta_{ig}$$

All predictors are standardized $\:(mean\:0,\:SD\:1)$.

Model 1 (Adolescents):

$$\mathrm{logit}\left({\upmu}_{\mathrm{adolescent},i}\right)={\beta}_{0}+{\beta}_{1}\cdot{ChildObesity}_{i}+{\beta}_{2}\cdot{AirPollution}_{10-19,i}+{\beta}_{3}\cdot{HealthSpending}_{i}+{\beta}_{4}\cdot{SportsFacilities}_{i}+{\beta}_{5}\cdot{WorkingHours}_{i}+{\beta}_{6}\cdot{GDPPercapita}_{i}+{\beta}_{7}\cdot{Urban}_{i}+{\beta}_{8}\cdot{Internet}_{i}$$

Model 2 (Adults, ages 18–69):

$$\mathrm{logit}\left({\upmu}_{\mathrm{adult},i}\right)={\beta}_{0}+{\beta}_{1}\cdot{AdultBMI}_{i}+{\beta}_{2}\cdot{AirPollution}_{20+,i}+{\beta}_{3}\cdot{Smoking}_{20+,i}+{\beta}_{4}\cdot{HealthSpending}_{i}+{\beta}_{5}\cdot{SportsFacilities}_{i}+{\beta}_{6}\cdot{WorkingHours}_{i}+{\beta}_{7}\cdot{GDPPercapita}_{i}+{\beta}_{8}\cdot{Urban}_{i}+{\beta}_{9}\cdot{Internet}_{i}$$

Model 3 (Older Adults, ages 70+):

$$\mathrm{logit}\left({\upmu}_{\mathrm{older},i}\right)={\beta}_{0}+{\beta}_{1}\cdot{AdultBMI}_{i}+{\beta}_{2}\cdot{AirPollution}_{70+,i}+{\beta}_{3}\cdot{Smoking}_{70+,i}+{\beta}_{4}\cdot{HealthSpending}_{i}+{\beta}_{5}\cdot{SportsFacilities}_{i}+{\beta}_{6}\cdot{WorkingHours}_{i}+{\beta}_{7}\cdot{GDPPercapita}_{i}+{\beta}_{8}\cdot{Urban}_{i}+{\beta}_{9}\cdot{Internet}_{i}$$

where $\:i$ indexes countries $\:(i\:=\:1,\:...,\:38)$, $y_{ig}$ is the proportion of the population meeting WHO physical activity guidelines (bounded between 0 and 1), $μ_{ig}$ is its expected value, $φ_g$ is the group-specific precision parameter, $\:\beta\:₀$ is the intercept, $\:\beta\:₁...\beta\:₉$ are regression coefficients for the respective predictors. Unlike ordinary linear regression, beta regression specifies the stochastic component directly through the Beta distribution rather than an additive error term; the conditional variance is a function of the mean, $\mathrm{Var}(y_{ig}) = \mu_{ig}(1 - \mu_{ig})/(1 + \varphi_{g})$. The logit link function is used to map the bounded outcome variable to the real line, consistent with beta regression methodology. For Model 2, when non-linear terms were statistically significant (as determined by ANOVA and diagnostic tests), orthogonal polynomial terms were included to capture cubic relationships while minimizing multicollinearity. All independent variables were standardized prior to analysis to enable direct comparison of effect sizes across predictors.

### Data preprocessing and data exploration

Before analysis, we undertook a multi-step data preprocessing process to ensure the analytic dataset was as complete and comparable as possible across the 38 OECD countries for 2022. First, all available data for key study variables—including age- and sex-specific PA rates, BMI, DALYs, health spending, and sports facility density—were compiled from international sources. To construct accurate national estimates of PA prevalence, we calculated weighted averages based on each country’s female and male population shares, thus reflecting the true demographic structure rather than relying on simple unweighted means. In cases where country-level data were missing or only partially available, we applied advanced imputation methods, including k-Nearest Neighbors, MissForest, and XGBoost algorithms, to estimate the missing values. The selection of imputation model was based on best fit, guided by cross-validation and predictive accuracy metrics. To further improve the validity of imputations, auxiliary variables such as the national male/female population distribution and NCD mortality rates were incorporated, since these variables are strongly associated with both the main predictors and outcomes. For variables with time series gaps (e.g., health spending), forecasting models were used. These steps were necessary to harmonize the data and avoid bias due to incomplete reporting, enabling robust cross-country comparisons. All technical details, model specifications, and diagnostic results for these procedures are presented in Supplementary Material Section S2.

### Sample sufficiency

Sample sufficiency measures how well inferences about a population can be drawn. According to the results of the power analysis conducted to determine the effect of the sample size (*n* = 38) used in the current study on the model power, the minimum effect size that can be determined at 80% statistical power and 5% significance level in the beta regression model with eight independent variables was calculated as f²=0.51 (corresponding to Model 1). It is also above the large effect size (f²=0.35) according to Cohen’s classification [74]. Accordingly, power analyses were examined separately according to the models. According to the results of Model 1, the effect size was calculated as f²=0.233 and remained below the expected level. The analysis with the effect size f²=0.66 calculated for Model 2 showed that the model with 11 independent variables had 83% statistical power with a sample size of 38 observations. This shows that the minimum effect size that can be determined at 80% power level is f²=0.62. Since the effect size (f²=0.66) is above this threshold, it indicates that the analysis produces reliable results and the risk of type II error is at an acceptable level. For Model 3, it was determined that the beta regression model has a very high statistical power. The analysis with the calculated effect size f²=0.714 revealed that the model with 9 independent variables has 91% statistical power with a sample size of 38 observations. It shows that the minimum effect size that can be determined at 80% power level with sample size and number of variables is f²=0.545. Since the effect size of the model (f²=0.714) is above this threshold, it is accepted that the analysis produces high reliability results.

### Statistical analysis

All analyses were conducted using R statistical software (version 4.4.2; R Core Team, 2023, Vienna, Austria). Prior to analysis, all independent variables were standardized to ensure comparability of effect sizes. For analytical procedures, the ‘car’ package from R packages was used for Variance Inflation Factor (VIF) analysis to assess multicollinearity between estimators [75]. We assessed the model specification and linearity assumptions (see Supplementary Material Section S2) with the ‘lmtest’ package and also used it for the Ramsey Regression Equation Specification Error Test (RESET) for model linearity [76]. Power analysis was performed to assess the adequacy of the sample size using the ‘pwr’ package [77]. The package ‘betareg’ was used to set up the beta regression model, modelling proportional data constrained between 0 and 1 [78]. For each age group, a separate beta regression model was constructed with the proportion of physically active individuals as the dependent variable. Linearity assumptions were probed by adding quadratic and cubic terms for each predictor; when a significant cubic pattern emerged (adult air-pollution DALYs), we first created the naïve quadratic and cubic terms but detected severe multicollinearity (VIF > 190). To resolve this, we generated orthogonal polynomials *(code: poly())*, after which the generalised VIF fell to 8.23 for the 3rd-order term and below 4 for all remaining predictors—well within acceptable limits. Statistical significance was interpreted at a 5% type I margin of error and extended to 10% for marginal significance interpretation. Model fit was interpreted using Pseudo R² values representing the proportion of variance explained by each model. Phi (φ) was used as a sensitivity parameter to assess the predictive accuracy and dispersion of the model.

## Results

**Fig. 1:**
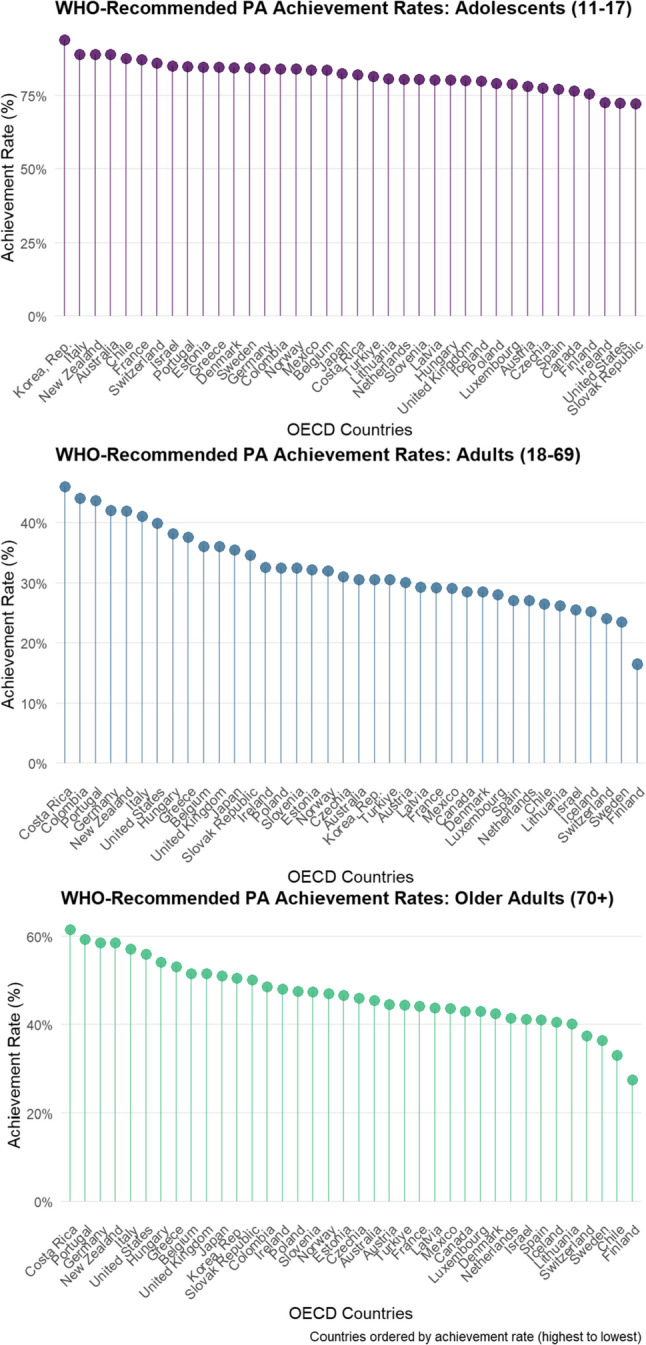
WHO-recommended PA achievement rates by age group in OECD countries (2022)

Figure 1 provides an overview of the percentage of the population in OECD countries meeting the WHO-recommended PA guidelines, disaggregated by age group.

Figure 1 provides an overview of the percentage of the population in OECD countries meeting the WHO-recommended PA guidelines, disaggregated by age group. Achievement rates are consistently highest among adolescents, with most countries exceeding 70% compliance; however, there is still observable variation, with some countries falling below this level. Among adults (18–69 years), the proportion achieving recommended PA drops markedly, with the majority of OECD countries reporting rates below 40%. The lowest achievement rates are observed in older adults (70+), where country averages cluster between 35% and 50%, and several countries fall below 40%. Notably, Costa Rica emerges as one of the highest-performing countries for both adult and older adult groups, while Finland appears at the lower end for these age groups.

The analysis includes three separate models: Model 1, which examines the determinants of PA among adolescents aged 11–17 years; Model 2, which investigates factors affecting PA among adults aged 18 to 69 years; and Model 3, which focuses on the drivers of PA in older adults aged 70 and above. Each model is tailored to reflect the age-specific health burdens and socioeconomic-environmental context relevant to the respective life stage. Detailed multicollinearity and linearity checks were performed for all models; results of these diagnostic tests (including VIF values, RESET, and Locally Estimated Scatterplot Smoothing [LOESS] visualizations) are reported in Supplementary Material Section S3.

The main results from Model 1, examining predictors of adolescent PA, are summarized in Table 2.

**Table 2:** Factors influencing adolescent PA

Model 1	β	SE	95% CI	z-value	*p*
*Intercept*	**1.528**	**0.049**	**[1.432**,** 1.624]**	**31.131**	2 × 10⁻¹⁶***
Childhood Obesity (BMI, Ages 10–18)	0.004	0.074	[− 0.142, 0.149]	0.048	0.9620
Air Pollution-Related DALYs (Ages 10–19)	−0.054	0.056	[− 0.164, 0.055]	−0.972	0.3312
Health Spending ($)	−0.008	0.065	[− 0.136, 0.120]	−0.126	0.8997
Access to Sports Facilities	−0.023	0.065	[− 0.150, 0.104]	−0.351	0.7258
Average Annual Working Hours	−0.106	0.093	[− 0.288, 0.077]	−1.133	0.2573
GDP per Capita	−0.036	0.084	[− 0.201, 0.128]	−0.434	0.6643
Urban Population Share (% of Total)	**0.119**	**0.055**	**[0.011**,** 0.227]**	**2.159**	0.0309*
Internet Penetration Rate (% of Population)	−0.157	0.099	[− 0.351, 0.037]	−1.589	0.1120
*Precision Model (φ)*	**74.12**	**16.95**	**[40.90**,** 107.34]**	**4.373**	1.23 × 10⁻⁵***

Dependent Variable: Adolescent PA Rate; Maximum Likelihood Estimator; Pseudo R²: 0.1891

*PA* Physical Activity, *BMI* Body Mass Index, *GDP* Gross Domestic Product, *DALYs* Disability-Adjusted Life Years, φ Precision parameter

*: *p* < 0.05; **:*p* < 0.001

According to the results of the beta regression analysis of Model 1, among the variables affecting the rate of adolescent PA, the urban population ratio (%) had a statistically significant and positive effect (*p* = 0.0309), while the other variables were not significant. Adolescent PA rate is higher in countries with higher urbanization rate. While the intercept of the model showed a very strong significance, the sensitivity parameter (phi) of the model was also statistically significant and supported the reliability of the model. Pseudo R² value (0.1891) showed that the explained variance of the model was 18.91% (Table 3). In the continuation of Model 1, the beta regression analysis in which the urbanization variable was used alone was also examined. In this sub-model, the variable lost its statistical significance (*p* = 0.222) and the explained variance decreased to 3.4%. This showed that the effect of urbanization on adolescent PA rate emerged only when other socioeconomic and environmental factors were controlled.

**Table 3:** Factors influencing adult PA with non-linear model

Model 2	β	SE	95% CI	z-value	*p*
*Intercept*	**-0.749**	**0.038**	**[-0.823**,** -0.675]**	**-19.791**	2 × 10⁻¹⁶***
Adult BMI (Ages 18–69)	0.014	0.051	[-0.086, 0.115]	0.279	0.7799
Air Pollution-Related DALYs (Ages 20+) *Linear Term*	-0.713	0.453	[-1.601, 0.175]	-1.575	0.1152
Air Pollution-Related DALYs (Ages 20+) *Quadratic Term*	0.026	0.299	[-0.561, 0.612]	0.085	0.9319
Air Pollution-Related DALYs (Ages 20+) *Cubic Term*	0.300	0.282	[-0.253, 0.853]	1.063	0.2879
Smoking-Related DALYs (Ages 20+)	0.063	0.054	[-0.043, 0.169]	1.169	0.2426
Healthcare Expenditure Per Capita	-0.026	0.065	[-0.154, 0.102]	-0.397	0.6916
Access to Sports Facilities	**0.172**	**0.073**	**[0.028**,** 0.316]**	**2.339**	0.0194**
Average Annual Working Hours	-0.036	0.061	[-0.156, 0.085]	-0.585	0.5585
GDP per Capita	0.034	0.069	[-0.101, 0.169]	0.496	0.6199
Urban Population Share (% of Total)	0.016	0.045	[-0.071, 0.104]	0.370	0.7117
Internet Penetration Rate (% of Population)	**-0.126**	**0.069**	**[-0.261**,** 0.009]**	**-1.828**	0.0676*
*Precision Model (φ)*	**84.20**	**19.22**	**[46.53**,** 121.87]**	**4.382**	1.18 × 10⁻⁵***

Dependent Variable: Adult PA Rate; Maximum Likelihood Estimator; Pseudo R²: 0.398

*PA* Physical Activity, *BMI* Body Mass Index, *GDP* Gross Domestic Product, *DALYs* Disability-Adjusted Life Years, φ Precision parameter

*: *p* < 0.1; **:*p* < 0.05; ***:*p* < 0.001

In Model 2 (adults), a significant non-linear (cubic) relationship was detected only for the air pollution-related DALY variable, which was therefore modeled using orthogonal polynomials to address multicollinearity and ensure stable coefficient estimates. All other predictors were included as linear terms. This approach was guided by diagnostic tests and ANOVA results; comprehensive methodological details and diagnostic plots are provided in Supplementary Material Section S3. The main results for Model 2 are summarized in Table 3.

According to the results of the analysis of the non-linear beta regression model applied to Model 2, among the factors affecting adult PA rate, access to sports facilities (β = 0.17157, *p* = 0.0194) had a statistically significant and positive effect, while internet penetration rate (β=-0.12588, *p* = 0.0676) showed a marginally significant and negative effect. None of the linear, quadratic and cubic terms of DALYs due to air pollution in adults were found to be statistically significant. Other factors such as adult BMI, DALY due to smoking, health expenditures, working hours, GDP per capita and urban population ratio did not show statistically significant effects. The intercept term (*p* < 0.001) and the sensitivity parameter (φ = 84.2, *p* < 0.001) of the model were highly significant and the Pseudo R² value of the model was 0.398, indicating that the model explained approximately 40% of the variance in the dependent variable (Table 3).

In Model 3, the relationship between the logit-transformed values of PA rates of adults over 70 years of age and all independent variables was found to be linear. In the ANOVA test results, quadratic or cubic terms were not found statistically significant for any variable. Therefore, in the beta regression model of Model 3, all variables were used in linear form (Table 4).

**Table 4:** Factors influencing PA in adults aged 70 and over

Model 3	β	SE	95% CI	z-value	*p*
*Intercept*	**-0.129**	**0.037**	**[-0.202**,** -0.057]**	**-3.504**	0.000458***
Adult BMI (Ages 18–69)	0.024	0.046	[-0.067, 0.115]	0.523	0.6012
Air Pollution-Related DALYs (Ages 70+)	**-0.128**	**0.058**	**[-0.243**,** -0.014]**	**-2.196**	0.02809*
Smoking-Related DALYs (Ages 70+)	**-0.097**	**0.044**	**[-0.183**,** -0.011]**	**-2.218**	0.02654*
Healthcare Expenditure Per Capita	-0.008	0.060	[-0.124, 0.109]	-0.128	0.8984
Access to Sports Facilities	**0.178**	**0.056**	**[0.068**,** 0.288]**	**3.183**	0.00146**
Average Annual Working Hours	-0.069	0.061	[-0.189, 0.051]	-1.127	0.2596
GDP per Capita	0.022	0.063	[-0.102, 0.146]	0.348	0.7280
Urban Population Share (% of Total)	0.002	0.043	[-0.082, 0.087]	0.050	0.9600
Internet Penetration Rate (% of Population)	**-0.143**	**0.066**	**[-0.273**,** -0.013]**	**-2.165**	0.03041*
Precision Model (φ)	**77.30**	**17.62**	**[42.77**,** 111.83]**	**4.387**	1.15 × 10⁻⁵***

Dependent Variable: Adult PA Rate; Maximum Likelihood Estimator; Pseudo R²: 0.4167

*PA* Physical Activity, *BMI* Body Mass Index, *GDP* Gross Domestic Product, *DALYs* Disability-Adjusted Life Years, φ Precision parameter

*: *p* < 0.05; **:*p* < 0.01; ***:*p* < 0.001

According to the results of Model 3 beta regression analysis, statistically significant effects of four variables were found among the factors affecting the PA rate of adults over 70 years of age. Access to sports facilities (β = 0.178, *p* = 0.001) had a strong positive effect, while DALYs due to smoking (β=-0.097, *p* = 0.027) showed a negative effect. Also, DALYs due to air pollution (β=-0.128, *p* = 0.028) and internet penetration rate (β=-0.143, *p* = 0.030) had a negative effect. Other factors such as adult BMI, health expenditures, working hours, GDP per capita and urban population ratio did not show statistically significant effects. The model’s intercept term (*p* < 0.001) and sensitivity parameter (φ = 77.30, *p* < 0.001) were highly significant, and the Pseudo R² value of the model was found to be 0.4167, indicating that the model explained approximately 42% of the variance in the dependent variable (Table 4).

## Discussion

Across OECD countries, the proportion of adolescents meeting WHO-recommended PA guidelines remains relatively high compared to adults and older adults, yet striking cross-country disparities are evident. Korea, New Zealand, and Ireland lead with adolescent PA achievement rates exceeding 85%, while the Slovak Republic and Greece show rates closer to 70%. In contrast, PA achievement among adults (18–69 years) drops sharply, with only Costa Rica, Colombia, and New Zealand approaching or surpassing 40%, while Finland and Switzerland report rates below 20%. The decline continues in older adults (70+), where Costa Rica and Portugal exceed 55%, but several European countries, including Finland and Sweden, fall below 35%. Understanding the factors behind these variations is critical for designing targeted interventions. Our findings reveal that urbanization, rather than being a standalone determinant, interacts with socioeconomic and environmental conditions to shape adolescent PA. Urban settings, when supported by well-planned infrastructure and recreational spaces, appear to foster higher activity levels, but without such enabling contexts, urbanization alone does not guarantee more active lifestyles. In a study by Ewing and Cervero (2010), it was observed that a one-unit increase in population density, diversity of use and design elements increased the likelihood of individuals walking and using public transport [55]. Martin et al. conducted a study in both urban and rural areas of the USA and concluded that the recreational PA levels of individuals living in urban areas were 15% higher than those living in rural areas, but the duration of PA was longer due to labour-based jobs in rural areas [56]. This suggests that the PA levels of adolescents living in urban areas are shaped in interaction with other factors in urban environments (health expenditures, technology use, sports facilities, etc.) rather than being an effect of urbanization alone. The importance of urban planning in promoting PA among adolescents is increasing day by day. Policies that aim to create walkable neighborhoods, safe parks and accessible recreation areas are expected to contribute to increasing the activity levels of young people living in urban areas.

Although PA served as the dependent variable in our models, its well-documented inverse correlation with disease burden provides contextual insight into its variation across diverse environmental and health conditions. Consistent with previous findings, a study of 129 children aged 9–14 years in New York reported that higher levels of PA were associated with a lower risk of airborne disease transmission [37]. Similar studies have observed correlations between PA and reduced health risks related to air pollution [38, 79]. However, high levels of air pollution are known to restrict outdoor activities, particularly among children and adolescents, due to health concerns. Additionally, both a separate study and a systematic review with meta-analysis found that air pollution increases physical inactivity among children and adults [29, 39]. PA has been consistently associated with lower mortality, lower risk for NCDs and improved mental health in observational studies [36].

Non-linear beta regression analysis analyzing the factors affecting PA levels of adults in OECD countries revealed that access to sports facilities (β = 0.172, *p* = 0.019) had a strong positive effect on PA. In contrast, internet penetration rate (β=-0.126, *p* = 0.068) was marginally associated with lower PA levels. These findings emphasize the importance of access to infrastructural facilities that encourage PA and suggest that the increasing use of technology in modern society, especially internet access, may reduce PA levels by encouraging sedentary lifestyles.

When the data on sports facilities are analyzed, it is proven that there is a significant positive relationship between sports facilities and PA rates. As a result of the systematic review published by Kaczynski and Henderson, it was reported that proximity to sports facilities has a positive effect on PA [59]. In addition, in a study conducted by Potwarka et al. in Canada, it was found that individuals living close to a sports facility were more likely to meet the WHO’s recommended exercise goal than those living further away [60]. In another study published by McCormack and Shiell, it was reported that there was a significant positive relationship between the presence and abundance of sports facilities and participation in PA [61].

The results of beta regression analyses of factors affecting PA in adults over 70 years of age showed that access to sports facilities (β = 0.178, *p* = 0.001) significantly increased PA in this age group, while environmental health risks revealed significant effects. Both air pollution-related health burden (β=-0.128, *p* = 0.028) and internet penetration rate (β=-0.143, *p* = 0.030) were found to negatively affect PA. In addition, smoking-related health burden (β=-0.097, *p* = 0.027) also had a negative effect, indicating that chronic health problems and deterioration of general health status may limit the PA capacity of elderly individuals. These findings suggest that while promoting PA in the elderly population, air quality as an environmental factor and smoking as a behavioral determinant, and the use of digital technology should be managed in balance. For older adults, policies that reduce air pollution and tobacco exposure, together with digital education to support healthy technology use, are critical to minimize barriers to PA. Urban and health policy-makers should consider multi-sectoral interventions targeting these environmental and behavioral obstacles. In the study published by Henchoz et al., it was reported that individuals over 65 years who used the internet engaged in 32 min more PA per week on average compared to those who did not use the internet [80]. This difference might reflect easier access to health-related information and resources through the internet among older adults. The easy access of the elderly individuals in the study to information related to PA through the internet was presented as the reason for this difference. In a study conducted in the USA and Thailand, Penglee et al. examined the duration of phone use and PA of university students and found that students who used smartphones for 4 h or more daily had 45 min less exercise time than those who used smartphones for less than 2 h [58].

When the studies on the triangle of air pollution, PA and health concept are analyzed, it is possible to reach similar results [30]. Kyra Naumoff Shields et al. found that exposure to traffic-related air pollutants caused acute changes in heart rate variability (HRV), especially gaseous pollutants such as ozone and formaldehyde decreased HRV, whereas PM2.5 exposure increased HRV parameters. In the same study, it was also stated that PA may play a role in mitigating these adverse effects [30]. Similarly, in a randomized, double-blind, crossover intervention study of healthy adults, Han et al. found that short-term exposure to traffic-induced air pollution (TRAP) had acute effects on heart rate, blood pressure and heart rate variability, and that filtering particulate matter could attenuate these effects to a limited extent; however, overall mask and filter interventions provided limited protection. This study showed that the effect of physical barriers was limited, but TRAP exposure had significant effects on the cardiovascular system [31].

D’Oliveira et al. it was reported that air pollution - especially particulate matter such as PM2.5 - reduces the favorable effects on health during PA in older people, while some studies reported that PA mitigates the negative effects of pollutants [32]. In a global modelling study by Tainio et al. it was shown that active modes of transport such as walking and cycling provide significant health benefits; however, in cities with very high air pollution levels, these benefits may be reduced and may turn into harm if certain thresholds are exceeded [33]. Similar to the present study, a direct negative relationship between air pollution and PA was found in the study by Xu et al. [34].

Several conceptually important covariates—BMI/obesity prevalence, average annual working hours, GDP per capita, and, in Models 2–3, urban population share—did not reach statistical significance and warrant cautious interpretation. The null results for BMI align with its well-known limitations: it conflates fat and lean mass, and validity varies by age, sex, and ethnicity [81, 82]. Conventional obesity thresholds (e.g., BMI ≥ 30; 27.5 kg/m² in some Asian populations) can be especially blunt in older adults, where sarcopenia and age-related shifts complicate risk classification [83, 84]. The bidirectional link between BMI and PA—lower PA promoting weight gain while higher BMI can constrain activity—also weakens cross-sectional associations in ecological designs [85]. National averages of working hours likely obscure within-country differences in schedule flexibility, occupation-specific physical demands, and work–life policies, reducing explanatory power [86]. The absence of a GDP effect suggests that wealth does not automatically translate into more active populations: greater resources may fund facilities but can coexist with automation, car dependence, and other sedentary conveniences [54, 87]. Urban population share was significant only for adolescents—and only when other contextual factors were controlled—indicating that the relevant mechanism is the quality and equity of the built environment rather than density per se [88]. These patterns point to the limits of nationally aggregated, cross-sectional data and the need for multilevel designs that combine individual behaviors with contextual determinants.

In the systematic review study conducted by Kilgour et al. it was tried to convey the barriers and motivations of individuals over 70 years of age regarding PA. In the study, factors affecting PA rates for adults over 70 years of age were found as weather conditions, financial factors, social environment support, self-efficacy, stress, having free time, facility adequacy [89]. Similar studies have concluded that the accessibility of sports facilities is an important source of motivation [90, 91]. All these findings reveal that holistic approaches, in which environmental, technological and structural factors are considered together in a multidimensional manner, should form the basis of sustainable public health strategies to increase PA.

## Limitations

The primary limitation of this study lies in its cross-sectional design, which prevents establishing temporal relationships and limits causal inference. Although secondary data from multiple international sources were harmonized, some residual inconsistencies may persist. Notably, data on access to sports facilities were missing for several countries, necessitating the use of ad hoc surveys and model-based estimations, which may have introduced additional uncertainty. Similarly, 2022 values for selected health and economic indicators were imputed using time-series forecasts. While data-driven, these estimates are predictive rather than observed, potentially affecting the precision of results. The relatively small sample of 38 OECD countries further constrains statistical power—especially in Model 1, where only large effect sizes could be reliably detected.

An additional limitation stems from the age-stratified ecological design. Key explanatory variables (e.g., GDP per capita, urban population share, and internet penetration) were measured at the national level and not disaggregated by age, limiting the granularity of age-specific interpretations. Moreover, slight inconsistencies exist between the age categories used for PA prevalence and those applied to certain explanatory variables (e.g., PA measured in ages 11–17 versus covariates referencing ages 10–18 or broader age groups). While this approach allows for macro-level analyses of how contextual factors shape PA prevalence across life stages, it may obscure within-country heterogeneity and introduces a potential risk of ecological fallacy.

Future studies should aim to integrate nationally harmonized microdata—such as household surveys, school-based assessments, or health registries—with contextual indicators to validate and refine these associations at finer spatial and demographic scales. Finally, certain potentially relevant variables—such as national PA policies, cultural attitudes toward exercise, or detailed metrics on recreational infrastructure quality—were not available in standardized or up-to-date formats across all countries, which may have limited the explanatory capacity of our models.

## Conclusion

This study’s age-stratified beta regression framework highlights that the determinants of PA in OECD countries are neither consistent across life stages nor strictly linear in effect. Among adolescents, PA increases with urban population share—but only when adjusted for other contextual factors—indicating that urban density alone does not guarantee active lifestyles and must be supported by enabling environments. For adults, access to sports facilities emerged as the most robust positive predictor, while higher internet penetration was marginally associated with reduced activity. In older adults, although sports facility access remained influential, its benefits were offset by cumulative health burdens from air pollution, tobacco use, and screen time.

Interestingly, the cubic (non-linear) relationship tested for adult air-pollution DALYs was statistically insignificant, suggesting the presence of a threshold or saturation point rather than a gradual dose-response pattern—an area warranting further exploration through flexible modelling techniques. Overall, the findings suggest that environmental exposures, behavioral technologies, and built-environment features interact uniquely with age-specific physiological and social factors. Consequently, public health interventions must be tailored to age, multisectoral in scope, and responsive to potential non-linear dynamics in PA determinants.

### Policy recommendations

For adolescents, the positive association between urbanization and PA underscores the importance of well-planned, compact urban environments. Urban planners should prioritize walkable neighborhoods, protected cycling lanes, safe school routes, and inclusive recreational areas. School administrators can complement these efforts by incorporating daily moderate-to-vigorous PA sessions and expanding after-school sports programs. Public health agencies could further support behavior change through exergaming interventions—digital applications that replace passive screen time with interactive physical activities designed to promote movement and engagement. These approaches can transform recreational screen use into opportunities for moderate-to-vigorous exercise, especially appealing to youth accustomed to digital environments.

For adults, enhancing access to sports facilities remains a key policy lever. Governments should prioritize equitable distribution of multipurpose fitness centers, particularly in underserved areas, through public-private partnerships and targeted subsidies. Employers can support workforce wellness through flexible schedules and fitness incentives. At the same time, public health campaigns should promote digital wellness and advocate for behavioral nudges that encourage movement amidst increasing screen time.

For older adults, policies must address the combined challenges of declining health, environmental exposures, and digital engagement. Age-friendly upgrades—such as low-impact equipment, safe walking paths, and subsidized senior fitness programs—should be paired with rigorous air quality and tobacco control measures. Digital inclusion efforts should promote tablet-based or virtual guided-exercise programs that encourage movement rather than sedentary screen use. A coordinated approach that integrates infrastructure, behavioral support, and environmental policy will be essential to promote active aging.

### Future research recommendations

Future research should prioritize the development of longitudinal, country-level panel datasets that track annual changes in PA prevalence alongside dynamic indicators such as sports facility density, pollution levels, and internet penetration. The application of flexible, non-linear causal inference techniques—including generalized additive models, Bayesian distributed-lag models, and synthetic control methods—will help uncover potential threshold effects and delayed responses that are not detectable in cross-sectional regressions.

Additionally, researchers should move beyond national averages to investigate disparities across gender, income, and regional contexts. Disaggregated analyses will illuminate hidden inequities and ensure that policy responses are both evidence-based and socially inclusive. Finally, integrating micro-level behavioral data with macro-contextual indicators will enhance explanatory power and inform more precise, context-specific policy interventions.

## Supplementary Information

Supplementary Material 1: Supplementary Material Section S1: Detailed variable definitions and the full list of all indicators included in each regression model, including sources and coding notes. Supplementary Material Section S2: Technical documentation of the data preprocessing, imputation procedures, and machine learning steps, including cross-validation results and sensitivity analyses. Supplementary Material Section S3: Model diagnostics for all regression models, including VIF, RESET, and LOESS visualizations of non-linearity.

## Data Availability

The original data presented in this study are openly available at https://zenodo.org/records/15126227, (accessed on 25 April 2025).

## Abbreviations

BMI: Body Mass Index
DALYs: Disability-Adjusted Life Years
GDP: Gross Domestic Product
IHME: Institute for Health Metrics and Evaluation
ITU: International Telecommunication Union
LOESS: Locally Estimated Scatterplot Smoothing
NCDs: Non-Communicable Diseases
OECD: Organisation for Economic Co-operation and Development
PA: Physical Activity
RESET: Regression Equation Specification Error Test
R: R Statistical Software
US$: United States Dollar
VIF: Variance Inflation Factor
WHO: World Health Organization
