# Supplementary material for: How environmental health burdens shape physical activity patterns: age-specific evidence from OECD countries
Source: BMC Public Health. 2026 Jun 4;26:2017. doi: 10.1186/s12889-026-27950-9 (PMC13326186; doi:10.1186/s12889-026-27950-9)
Supplement: Supplementary file 1 — Supplementary Material 1: Supplementary Material Section S1: Detailed variable definitions and the full list of all indicators included in each regression model, including sources and coding notes. Supplementary Material Section S2: Technical documentation of the data preprocessing, imputation procedures, and machine learning steps, including cross-validation results and sensitivity analyses. Supplementary Material Section S3: Model diagnostics for all regression models, including VIF, RESET, and LOESS visualizations of non-linearity. [file 12889_2026_27950_MOESM1_ESM.docx]

**Supplementary Material**

**Section S1: Variable selection, conceptual rationale and variable included in each regression model**

This supplementary file provides a comprehensive overview of the data imputation and preprocessing procedures for the main study, *“Determinants of Physical Activity Levels Across Different Age Groups in OECD Countries.”* While the main text summarizes these steps due to space constraints, Supplementary material details the inclusion of each variable in the age-stratified regression models, as well as the role of auxiliary variables used exclusively for data imputation and harmonization.

**Variable Selection and Conceptual Rationale**

This study is based on the most recent records and aims to understand the determinants of PA levels across various age groups using a range of explanatory variables. We selected a set of socioeconomic, environmental, and health-related variables that are both theoretically relevant and empirically supported as influential drivers of PA. In our models, the dependent variables are the prevalence rates of PA for three different age groups, operationalized as the proportion of the population meeting the World Health Organization PA recommendations: at least 60 minutes of moderate-to-vigorous PA daily for adolescents (ages 11–17), and at least 150 minutes of moderate-intensity activity per week (or equivalent) for adults (ages 18–69) and older adults (ages 70 and above) (WHO, 2022: Global status report on PA 2022). The independent variables examined as explanatory factors include age-specific BMI; air pollution-related DALYs and smoking-related DALYs per 100,000 population; healthcare expenditure per capita (in current US dollars); access to sports facilities per 100,000 population; average annual working hours; Gross domestic product (GDP) per capita (in current US dollars); urban population share (as a percentage of total population); and internet penetration rate (percentage of the population with internet access). In addition, three auxiliary variables—the share of the male and female population, and the national NCDs mortality rate—were not included as independent variables in the regression models, but were used exclusively for data preprocessing and imputation procedures. Specifically, these variables supported the generation of demographically weighted and plausible national estimates of age- and sex-specific PA prevalence, especially for countries with incomplete or missing data. Detailed descriptions of all variables and their corresponding secondary data sources are provided in Table S1 [1–13].

**Table S1.** Research Variables and Sources

| **Variables** | **Definition** | **Time** | **Source** | **Link** |
| --- | --- | --- | --- | --- |
| PA Prevalence (%) | Proportion of population meeting WHO PA guidelines (by age and sex) | 2022 | WHO | <https://www.who.int/teams/health-promotion/physical-activity/global-status-report-on-physical-activity-2022> |
| Mortality Rate from NCDs (%) | Mortality rate from NCDs (Cardiovascular, diabetes, cancer etc.) | 2022 | WHO | <https://www.who.int/data/gho/data/themes/topics/topic-details/GHO/ncd-mortality> |
| GDP per capita (current United Stated Dollar) | Average income per capita (current US$) | 2022 | World Bank | <https://data.worldbank.org/indicator/NY.GDP.PCAP.CD> |
| Prevalence of Obesity among Adults | BMI ≥ 30 (crude estimate) (%) | 2022 | WHO | <https://www.who.int/data/gho/data/indicators/indicator-details/GHO/prevalence-of-obesity-among-adults-bmi–30-(crude-estimate)-(-)> |
| Prevalence of Obesity among Children and Adolescents | Prevalence of obesity among children and adolescents, BMI > +2 standard deviations above the median (crude estimate) (%) | 2022 | WHO | <https://www.who.int/data/gho/data/indicators/indicator-details/GHO/prevalence-of-obesity-among-children-and-adolescents-bmi-2-standard-deviations-above-the-median-(crude-estimate)-(-)> |
| Urban Population | Urban population (% of total population) | 2022 | World Bank | <https://data.worldbank.org/indicator/SP.URB.TOTL.IN.ZS> |
| Health Spending per capita (current United Stated Dollar) | Current health expenditure per capita (current US$) | 2000-2021 | World Bank | <https://data.worldbank.org/indicator/SH.XPD.CHEX.PC.CD> |
| Hours Worked Annual | Average annual hours actually worked per worker | 2010-2022 | OECD | <https://www.oecd.org/en/data/indicators/hours-worked.html?oecdcontrol-d7f68dbeee-var3=2023> |
| Internet penetration rate | Individuals using the Internet (% of population) | 2022 | ITU | <https://datahub.itu.int/data/?e=1&i=11624> |
| Air Pollution | 10-19 years old, 20+ years old, 70+ years old, Global Burden of Disease Study 2021 (GBD 2021) Results | 1990-2021 | IHME | <https://vizhub.healthdata.org/gbd-results/> |
| Smoking | 10-19 years old, 20+ years old, 70+ years old, Global Burden of Disease Study 2021 (GBD 2021) Results | 1990-2021 | IHME | <https://vizhub.healthdata.org/gbd-results/> |
| Population, Male (% of total population) | The percentage of the total national population that is male. | 2022 | World Bank | <https://data.worldbank.org/indicator/SP.POP.TOTL.MA.ZS?end=2023&name_desc=false&start=1960&view=chart> |
| Population, Female (% of total population) | The percentage of the total national population that is female. | 2022 | World Bank | <https://data.worldbank.org/indicator/SP.POP.TOTL.FE.ZS?end=2023&name_desc=false&start=1960&view=chart> |

Given that the explanatory variables (e.g., GDP per capita, urban population %, internet penetration) are measured at the national level, the present analysis adopts an age-stratified ecological approach. This means that age-specific PA prevalence rates are modeled as a function of broader country-level conditions. Such macro-contextual modeling is suitable when individual-level or age-disaggregated covariates are unavailable but cross-country comparisons are still of interest. The aim is not to infer within-country individual-level associations but rather to identify population-level patterns across OECD countries. To support this interpretation, we conducted robustness checks, including cluster-mean centering and intraclass-correlation diagnostics, which indicated that a significant proportion of variation in PA rates is attributable to between-country differences.

**GDP per Capita**

GDP per capita is widely recognized as a fundamental socioeconomic determinant of population-level PA. Higher national income typically enables greater public and private investment in sports infrastructure, active transportation systems, and health promotion programs, thereby facilitating increased opportunities for PA. Empirical studies have reported a positive correlation between GDP per capita and PA participation across countries. For instance, Rusesji and Maresova [14] found that higher GDP per capita was significantly associated with greater individual PA participation in an analysis involving 34 countries. However, at the macro level, a recent systematic review in public health field [15] highlighted that economic growth is often accompanied by shifts in occupational structures—from manual labor to more sedentary jobs—which can reshape leisure-time PA and, in some cases, lead to an overall decline in total activity even as national income rises. While increased resources can expand access to exercise facilities and structured activity, they may also reduce incidental or occupational PA, particularly in high-income OECD countries. These complex dynamics underscore the need to examine the differentiated effects of GDP per capita across age groups. In our study, we specifically investigate whether the facilitative role of greater economic resources in providing activity-supportive infrastructure outweighs the potential risk of increased sedentary behavior, especially among working-age and older adults. Furthermore, for adults aged 70 and above, GDP per capita may be especially relevant by enhancing access to community centers and programs that support active aging.

**Urban Population Share**

Urbanization acts as a double-edged sword in shaping PA patterns at the population level. On one hand, higher urban population share is often associated with increased availability of parks, sidewalks, and recreational facilities, all of which can facilitate active lifestyles—particularly among adolescents and young adults, who may benefit most from walkable environments and organized sports opportunities [16, 17]. On the other hand, rapid urban growth frequently brings challenges such as traffic congestion, elevated air pollution, and shrinking green spaces, which may deter outdoor activity and reduce overall PA levels [18]. Moreover, urban settings may expose residents to greater safety concerns and social inequalities, potentially compounding barriers to PA. This complex and sometimes contradictory influence of urbanization on PA highlights the importance of modeling urban population share as a central contextual variable in cross-national analyses. Our study, therefore, examines both the enabling and constraining aspects of urbanization in relation to age-specific PA prevalence, providing new evidence on how city environments shape active behavior across the life course.

**Internet Penetration Rate**

The rapid proliferation of digital technologies and the increasing prevalence of internet access have fundamentally reshaped daily life, affecting not only work and social interactions but also leisure-time behaviors [19]. Higher internet penetration is consistently linked to increased screen time and more widespread sedentary activities, contributing to lower levels of PA—a pattern that is especially pronounced among children, adolescents, and young adults [19, 20]. However, digitalization is not uniformly detrimental: for older adults and individuals with mobility limitations, internet access can facilitate engagement in online exercise programs and virtual communities that support active lifestyles. Despite these emerging opportunities, empirical research investigating the dual impact of internet penetration on PA across different age groups remains limited, particularly in high-income contexts. By considering internet penetration as a core variable, our study adds novel, age-stratified evidence to the literature on the complex relationship between digitalization and PA behaviors in OECD countries.

**Access to Sports Facilities**

The availability and accessibility of sports and recreational facilities are widely recognized as critical environmental determinants of PA. Numerous studies and meta-analyses have demonstrated a strong positive association between proximity to sports infrastructure—such as gyms, parks, and community centers—and increased PA participation across children, adolescents, adults, and older adults [21–25]. Easy access to safe and affordable facilities not only promotes routine exercise but also fosters social connectedness and community engagement [24, 25]. However, inequalities in the distribution and quality of these facilities can further exacerbate existing disparities in PA, particularly among socioeconomically disadvantaged or rural populations [24]. Recognizing these dynamics, our study [26] considers access to sports facilities as a central variable and, uniquely, incorporates a comprehensive data effort to harmonize and estimate facility density across OECD countries. In doing so, we aim to clarify the enabling or constraining role of facility access in shaping PA behaviors throughout the life course.

**BMI and Obesity Prevalence**

BMI and obesity prevalence serve as important indicators of nutritional status and overall health at both the individual and population levels. The relationship between BMI and PA is inherently bidirectional: high BMI can be both a consequence of low PA and a contributing factor to further inactivity, and the nature and strength of this association often vary by age group [27, 28]. In children and adolescents, rising obesity prevalence is typically regarded as a marker of declining PA and unhealthy lifestyle patterns, serving as an early warning signal for chronic disease risk. Among adults, given the complex interplay between work routines, leisure-time activity, and dietary habits, BMI can act as both a confounder and an effect modifier in the relationship between socioeconomic factors and PA. In older adults, excess body weight may further limit mobility and the ability to participate in regular exercise. To accurately capture these nuanced, age-specific dynamics, our study incorporates age-stratified BMI measures, enabling us to disentangle and test the independent and combined effects of body composition and PA across the life course.

**Air Pollution-Related DALYs**

Exposure to ambient air pollution—such as fine particulate matter (PM2.5) and ozone—has increasingly been recognized as a significant barrier to outdoor PA, particularly in densely populated urban environments [29–34]. Poor air quality can deter individuals from engaging in outdoor exercise due to health concerns, while chronic exposure has been linked to adverse cardiovascular and respiratory outcomes [30–32]. Importantly, air pollution not only restricts daily activity but also contributes to the broader burden of NCDs, an impact that is systematically measured by DALYs [29, 33, 34]. The negative effects of air pollution on PA are especially pronounced among vulnerable populations, such as children, older adults, and those with pre-existing health conditions. By incorporating age-specific air pollution-related DALY variables—Air Pollution-Related DALYs for Ages 10–19, Ages for 20-70, and Ages 70 and above— into our models, we aim to capture both the direct limitations imposed by poor air quality and the indirect, long-term health consequences that shape PA patterns across the life course. We believe this approach enables a more comprehensive assessment of environmental determinants in explaining PA disparities within and between countries.

**Smoking-Related DALYs**

Tobacco use remains one of the most significant modifiable risk factors for the development of chronic diseases, functional limitations, and premature mortality, particularly among older adults. Elevated levels of smoking-related DALYs capture both the immediate and cumulative health consequences of tobacco exposure at the population level [35, 36]. While tobacco use may have a relatively limited impact on PA among adolescents and young adults in the early stages of exposure, its adverse effects become increasingly pronounced as age and duration of smoking increase [35]. With cumulative exposure, smoking-related morbidity—including cardiovascular, respiratory, and oncological conditions—can substantially reduce PA capacity by diminishing functional health and endurance, particularly in older adults. By including age-specific smoking-related DALY variables in our analysis, we are able to account for the broader health-burdened environment in which PA behaviors take place and to better understand how tobacco-related disease burden may constrain or modify patterns of PA across different age groups.

**Healthcare Expenditure per Capita**

Per capita healthcare expenditure is commonly used as a proxy for the capacity, quality, and accessibility of a country's health system. Higher levels of health spending can foster environments that support PA by enabling broader public health campaigns, preventative health services, community-based exercise programs, and rehabilitation initiatives—all of which can raise awareness and lower barriers to PA across the population [37–40]. In countries with robust health investment, such initiatives may increase early detection of inactivity-related health risks and promote interventions to encourage active lifestyles. However, the impact of health expenditure on PA is not always straightforward. Differences in national spending priorities, health policy frameworks, and the efficiency with which resources are allocated can result in varied outcomes: increased spending may not always translate into greater participation in PA if investments are not directed toward preventive or health-promoting activities [37–39]. By including per capita healthcare expenditure in our models, we aim to capture not only the direct financial investment in health, but also the broader institutional capacity that can shape PA behaviors at the population level.

**Average Annual Working Hours**

Time constraints are among the most consistently reported barriers to regular PA, especially for working-age adults balancing employment and family responsibilities. National averages of annual working hours not only reflect individual time availability but also capture broader cultural norms, economic pressures, and labor market structures that shape daily routines. In contexts where average working hours are high, individuals may have fewer opportunities or less motivation to engage in leisure-time PA, contributing to higher rates of inactivity and associated health risks. Conversely, countries with shorter average working hours or more flexible work arrangements may facilitate greater participation in exercise and recreational activities [41–43]. Including average annual working hours in our analysis allows us to assess how structural and cultural factors related to work influence PA behaviors across different age groups and socioeconomic contexts.

**Use of Auxiliary Variables in Data Preprocessing and Imputation**

This study employed several auxiliary variables—specifically, the population shares of females and males, and the Mortality Rate from NCDs (%)—not as direct predictors in the main PA models, but rather as crucial inputs during data preprocessing and imputation steps.

The World Health Organization’s Global status report on PA 2022 presents PA rates separately for females and males [44]. However, for cross-country analysis and for countries with missing age- and sex-specific prevalence data, we required a unified, national-level estimate. To achieve this, we used each country’s 2022 female and male population shares as weights, producing a weighted average PA rate that accurately reflects the true demographic structure. This approach ensures that the aggregate estimates are not biased by countries’ varying sex distributions, which is especially important when imputing missing or partially reported data. The NCDs mortality rate variable was used exclusively during the data imputation and machine learning phases. Its inclusion was based on the strong epidemiological relationship between PA and NCDs mortality—both routinely reported in the same global datasets, and repeatedly shown in the literature to be tightly linked at the population level [45, 46]. Low PA prevalence is consistently associated with increased rates of NCDs mortality, including cardiovascular disease, diabetes, and certain cancers. In this context, the NCDs mortality rate serves as a robust auxiliary variable that improves the prediction of missing PA and health indicator values, especially in machine learning algorithms such as k-NN, MissForest, and XGBoost. Additionally, in some steps, this variable also served as a target outcome to validate the plausibility of our imputed estimates, given its established association with behavioral risk factors at the country level. This methodological decision is grounded in best practices from epidemiological and statistical research, which recommend the use of auxiliary variables that are epidemiologically or demographically relevant for imputing missing data and minimizing bias [47]. By integrating sex composition and NCDs mortality as auxiliary information—rather than as direct covariates in our regression models—we ensured that our final analyses were conducted on a maximally complete and demographically representative dataset, in line with the known interplay between PA and chronic disease burden. This strategy ultimately enhanced both the validity and generalizability of our cross-national findings.

**Variables Included in Each Regression Model**

The table below summarizes the independent variables included in each of the three age-stratified beta regression models. Model 1 targets adolescents (ages 11–17), Model 2 targets adults (ages 18–69), and Model 3 targets older adults (ages 70+). Each model includes a core set of socioeconomic, health, and environmental predictors, with additional age-specific health burden variables as appropriate (Table S2).

Table S2. Variables included in each regression model

| **Variables** | **Model 1 (Adolescents)** | **Model 2 (Adults)** | **Model 3 (Older Adults)** |
| --- | --- | --- | --- |
| Physical activity (age group) | ✓ | ✓ | ✓ |
| BMI / Obesity | ✓ (child BMI 10-18) | ✓ (adult BMI 19+) | ✓ (adult BMI 19+) |
| Air pollution-related DALYs | ✓ (10-19) | ✓ (20+) | ✓ (70+) |
| Smoking-related DALYs |  | ✓ (20+) | ✓ (70+) |
| Healthcare expenditure per capita | ✓ | ✓ | ✓ |
| Access to sports facilities | ✓ | ✓ | ✓ |
| Average annual working hours | ✓ | ✓ | ✓ |
| GDP per capita | ✓ | ✓ | ✓ |
| Urban population share | ✓ | ✓ | ✓ |
| Internet penetration rate | ✓ | ✓ | ✓ |

***Note:*** *The auxiliary variables—sex composition, national population, and NCD mortality rate—were not included as predictors in any of the regression models, but were used solely for data preprocessing and imputation steps, to enhance the demographic accuracy and validity of national estimates.*

**Use of Auxiliary Variables for Imputation and Harmonization**

In addition to the variables included in the main regression models, several auxiliary variables were used exclusively during the data preprocessing and imputation stages:

- Sex composition (male/female share, % of total population): Used only to compute demographically weighted national physical activity rates from age- and sex-specific prevalence data, particularly in cases where countries reported missing values by sex.
- Population (total, male, female): Utilized to harmonize prevalence rates and to ensure weighted aggregation across demographic strata.
- NCD mortality rate (%): Employed as a strongly associated auxiliary predictor in machine learning-based imputation models (e.g., k-NN, MissForest, XGBoost) for physical activity prevalence and health indicators, but not included as a direct covariate in regression models.

These auxiliary variables improved the validity and demographic representativeness of the imputed dataset, but were not used as predictors in the final regression analyses.

**Section S2: Data Imputation and Preprocessing Details**

To ensure a complete and harmonized cross-sectional dataset for 2022 across all 38 OECD countries, we sought to identify, recover, or estimate any missing values for key health indicators before commencing analysis. Specifically, missing data were present for age- and sex-specific physical activity prevalence rates—namely, Adult 18–69 Male, Adult 18–69 Female, Adult 70+ Male, Adult 70+ Female, Adolescent Male, and Adolescent Female. For Iceland and Israel, data were missing for all adult age/sex categories, while for Japan, the adolescent categories were incomplete. To address these gaps, we compared three machine learning algorithms—k-Nearest Neighbours (k-NN), Random Forest-based MissForest, and XGBoost—to impute missing values. Model performance was evaluated using Mean Absolute Error (MAE), Root Mean Square Error (RMSE), and R² metrics. As a preliminary step, we generated a correlation matrix to identify the variables most strongly associated with the missing data, thereby informing the selection of predictors for the imputation models (Fig. S1).


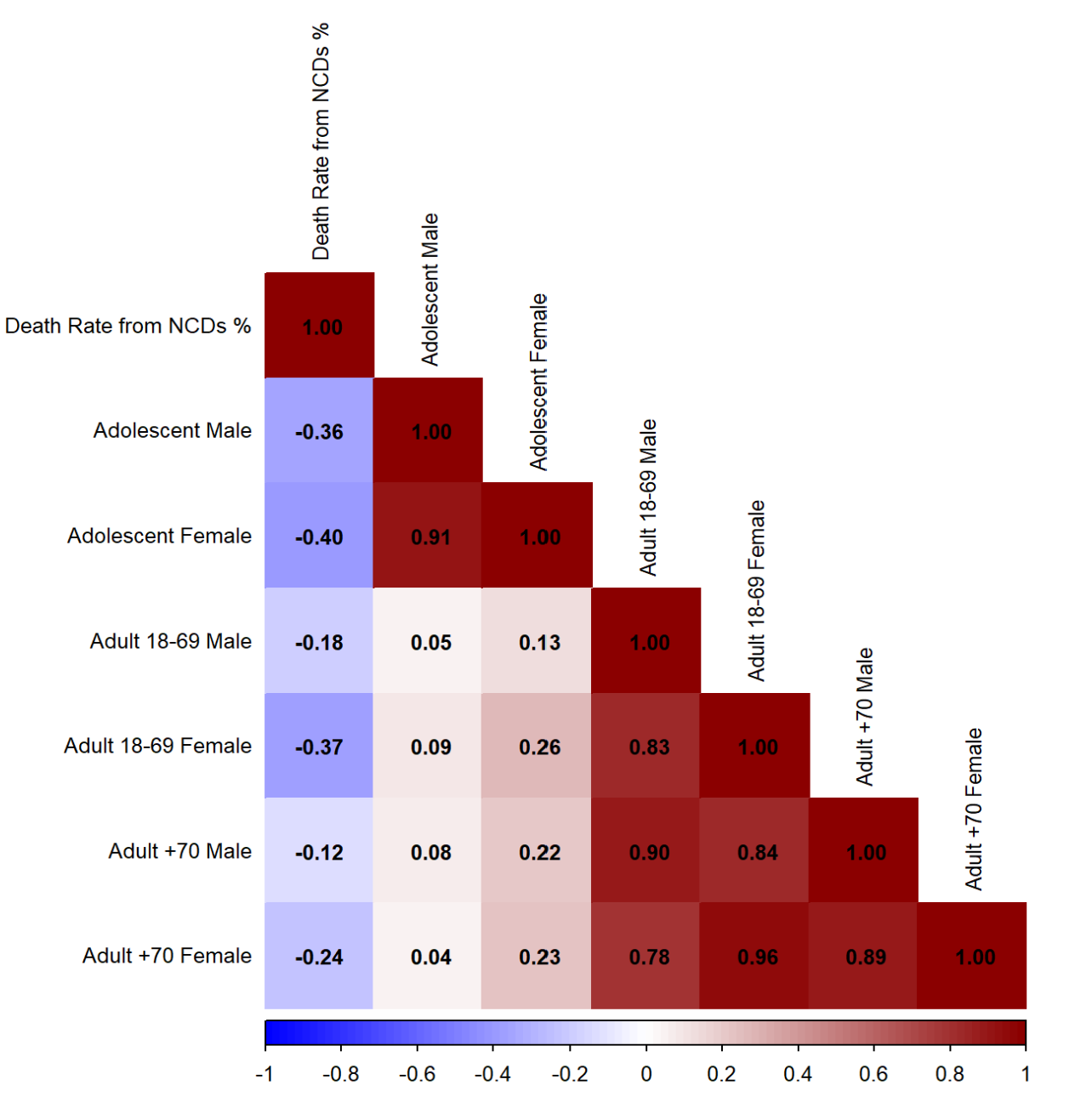


**Fig S1.** Correlation Between Death Rate from NCDs and Age-Specific Physical Activity Rates

The variable death rate from NCDs (Deaths due to Non-Communicable Diseases) was selected for use in machine learning models due to its strong association with age-specific physical activity levels. In some cases, it was used as an independent variable to estimate physical activity prevalence; in others, it was treated as a dependent variable, to explore how physical activity and other predictors influence the burden of disease. One of the main reasons for including this variable is that it is reported together with physical activity rates in the same database (Global status report on physical activity, WHO, 2022) and in the same study. In addition, the potential relationship between physical activity level and date rate from NCDs (%) makes it meaningful to include this variable in machine learning models. Many studies in the literature show that inadequate physical activity is one of the main risk factors of NCDs such as cardiovascular diseases, type 2 diabetes and some types of cancer [18, 36, 48–51]. For this reason, when physical activity levels are considered according to age groups, we thought that analysing how they are related to date rate from NCDs would enable us to make better predictions not only with the relationship with physical activity levels according to age groups, but also with date rate from NCDs values in the dependent variable position, and we included it in machine learning algorithms.

First, the data were evaluated with the KNN algorithm by scaling the data with 3, 5, 7 and 11 nearest neighbors for each missing value and the recommendation of using 5 nearest neighbors was determined, but this method also showed low accuracy in some variables. For this reason, we switched to the Random Forest based MissForest method, and again the algorithm was run by applying scaling, but although it gave more stable results in some variables, it again showed insufficient performance in some variables. Finally, for the XGBoost model, which is a robust model that does not need scaling, hyperparameter optimisation was performed repeatedly to minimise the risk of overfitting in the variables in question and to increase the generalisation success of the model (*Overfitting refers to a model learning the training data too closely, including noise, and thus performing poorly on new, unseen data. Overlearning, similar to overfitting, describes a situation where the model “memorizes” patterns in the training data, failing to generalize to other datasets*). In order to prevent XGBoost from overlearning, the maximum depth was reduced (max_depth=1), the learning rate was reduced (eta=0.1), the Lasso and Ridge penalties L1 (alpha=30) and L2 (lambda=50) were increased with a focus on Elastic Net regression, and the subsampling strategy was applied with a ratio of 70% to 30% (subsample=0.7, colsample_bytree=0.3). Thus, the model was ensured to make realistic predictions and not to memorise. Nevertheless, variables in which overlearning occurred were excluded when they remained above 95% variance explanation value.

**Table S3.** Comparison of Machine Learning Model Results and Model Selection

| **Variable** | **k-NN** | | | **missForest** | | | **XGBoost** | | | **Model Selection** |
| --- | --- | --- | --- | --- | --- | --- | --- | --- | --- | --- |
|  | **MAE** | **RMSE** | **R^2^** | **MAE** | **RMSE** | **R^2^** | **MAE** | **RMSE** | **R^2^** |  |
| **Adult 18-69 Male** | 3 | 3.536 | 0.907 | 1.25 | 1.658 | 0.937 | 1.422 | 1.484 | 0.851 | XGBoost |
| **Adult 18-69 Female** | 4.75 | 5.545 | 0.857 | 2 | 2.550 | 0.950 | 1.964 | 2.318 | 0.749 | XGBoost |
| **Adult +70 Male** | 4 | 4.243 | 0.458 | 3 | 4.301 | 0.796 | 3.523 | 4.139 | 0.995 | MissForest |
| **Adult +70 Female** | 5 | 6.892 | 0.703 | 1.75 | 2.179 | 0.550 | 1.289 | 1.488 | 0.884 | XGBoost |
| **Adolescent Male** | 4 | 4.243 | 0.893 | 5 | 5.788 | 0.020 | 0.467 | 0.735 | 0.999 | k-NN |
| **Adolescent Female** | 2.75 | 3.708 | 0.014 | 3 | 3.240 | 0.472 | 3.424 | 4.061 | 0.793 | XGBoost |

As a selection criterion for model selection, the models with the lowest MAE and RMSE values and R² values below 0.95 were preferred. According to this evaluation, XGBoost provided the best results for Adult 18-69 Male, Adult 18-69 Female, Adult +70 Female and Adolescent Female variables. However, since XGBoost was found to be overfitting in the Adult +70 Male variable, MissForest was selected as the best model for this variable. Similarly, XGBoost was found to be overlearning in Adolescent Male, so k-NN method was used as the best model. Adolescent Male is 79 for Japan; Adult +70 Male value for Iceland is 41,82166667 and 45.2 for Israel; Iceland 22,786 and Israel 22,864 for Adult 18-69 Male variable; Iceland 27,713 and Israel 28,216 for Adult 18-69 Female variable; Iceland 35,839 and Israel 36,190 for Adult +70 Male; Iceland 45,549 and Israel 46,268 for Adult +70 Female; and Japan 85,886 for Adolescent Female (Table S3).

While health spending, air pollution and smoking values are until 2021, it is observed that only some countries have 2022 values for health spending. Accordingly, Holt's Linear Trend and Holt's Detrended Trend models were used for forecasting based on their own past values from time series models for the values remaining in the gap:

$$l_{t}=\alpha*y_{t}+\left( 1-\alpha\right)*(l_{t-1}+b_{t-1})$$

$$b_{t}=\varphi*\left( l_{t-1}+b_{t-1} \right)+(1-\varphi)b_{t-1}$$

$$\hat{y}_{t+h|t}=l_{t}+h*b_{t}$$

$$and for detrented model:$$

$$y_{t}^{*}=y_{t}-\gamma_{t}$$

$$l_{t}^{*}=\varsigma*y_{t}^{*}+\left( 1-\varsigma\right)*(l_{t-1}+b_{t-1})$$

$$b_{t}=\beta*\left( l_{t-1}+b_{t-1} \right)+(1-\beta)b_{t-1}$$

$$\hat{y}_{t+h|t}^{*}=l_{t}^{*}+h*b_{t}^{*}$$

$$\hat{y}_{t+h|t}^{*}=l_{t}^{*}+h*b_{t}^{*}+\xi(t+h)$$

Missing data on health spending are forecast for countries using Holt's Linear Trend and Holt's Detrended Trend methods. All forecasts for health spending refer exclusively to missing 2022 values.


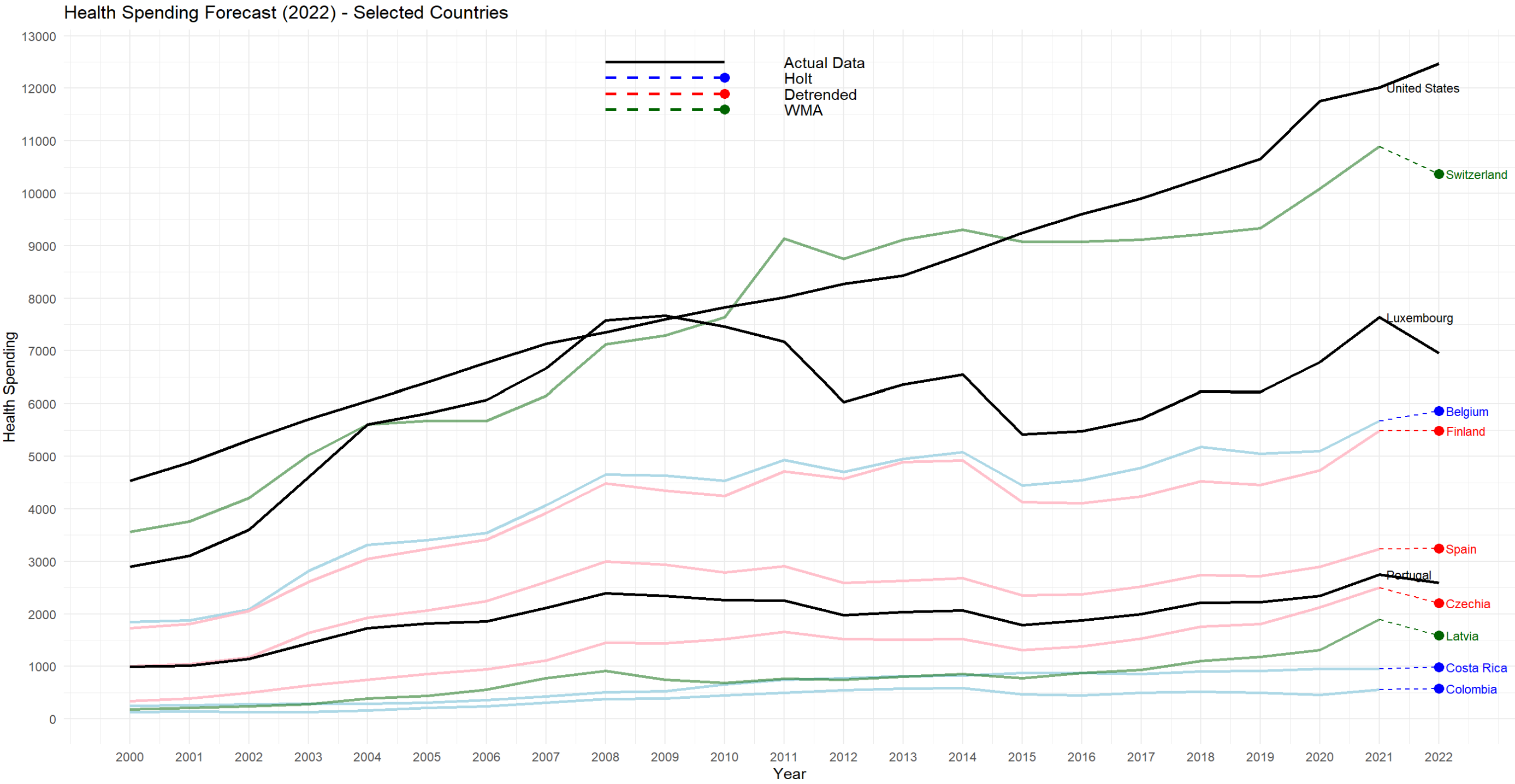


**Fig S2**. Health Spending Forecast for Selected Countries

The forecasts were evaluated with Mean Absolute Error (MAE), Mean Absolute Proportional Error (MAPE) and the correct method was tried to be selected. According to the comparison between the methods, Holt's Linear method was determined as a more reliable forecasting method since it showed lower MAE (95.87 - 2865.80) and MAPE (24.80 - 113.57) values for Australia, Austria, Belgium, Israel, Mexico, Netherlands, New Zealand and Türkiye. For Colombia, Costa Rica, Czech Republic, Spain, Finland, France, Greece, Hungary, Japan and Slovak Republic, the Detrended method is preferred as it offers lower error rates in the range of MAE (257.60 - 1595.51) and MAPE (27.08 - 124.55). Since the MAE (3731.04 - 3740.50) and MAPE (66.43 - 319.01) values of both methods are quite high in countries such as Switzerland and Latvia, alternative estimation methods had to be evaluated for these countries. In this context, the 3-year weighted moving average (WMA) forecast for Switzerland was calculated as 10,367.39, the MAE value was 244.79 and the MAPE value was 3.39%, indicating that the error rate of the model was low. For Latvia, the WMA forecast for 2022 is 1,583.83, MAE value is 66.72 and MAPE value is 8.10 per cent. Although the error rate for Latvia is higher than Switzerland, the value is accepted as it provides a reasonable estimate. In addition to the estimates for the burden of disease due to air pollution per 100,000 people aged 10-19 years using the same three methods, estimates for 2022 were made using 1990-2021 data for adults aged 20-69 years and older adults aged 70 years and over. Likewise, the burden of disease due to smoking was estimated separately for each country by applying prediction models for the variables for adults aged 20-69 years and older adults aged 70 years and over. (Fig S2).

Although 2022 forecasts were generated for the dataset, it was necessary to estimate the missing values for Poland’s burden of disease due to air pollution (DALY) for individuals aged 20–69 years and those aged 70 years and older. To address this, we employed a two-stage regression imputation approach. Prior to modeling, variable selection was guided by the results of a correlation matrix analysis. Specifically, we observed a moderate positive correlation between air pollution DALYs and smoking DALYs among adults aged 20–69 years (r = 0.527), and a very strong correlation between air pollution DALYs for individuals aged 70+ years and those aged 20–69 years (r = 0.936). These correlation coefficients informed our selection of dependent and independent variables within the imputation framework, maximizing predictive accuracy and model robustness.

In the first stage, the burden of disease due to air pollution for adults aged 20–69 years was estimated using smoking DALYs as the predictor. While smoking DALYs were statistically significant (p < 0.001), the model’s explanatory power, as measured by the coefficient of determination (R² = 0.2776), was relatively low. In the second stage, the estimated air pollution DALYs for the 20–69 age group were used to predict air pollution DALYs for those aged 70 and over. These two variables showed a very strong relationship (r = 0.936), which considerably increased the prediction accuracy; the model was highly significant (p < 0.0001) with high explanatory power (R² = 0.8755). As a result, the estimated air pollution DALYs for Poland were 1,374.6 for adults aged 20–69 years and 5,250.2 for those aged 70 and above.

According to OECD data, only Türkiye had missing data for the variable ‘Hours Worked Annual’ for the year 2022. In order to overcome this deficiency, forecasting was performed with four different time series models: Holt, SES, ETS and ARIMA. MAE and MAPE values of the models were calculated over the test data for 2021, and it was determined that the SES (MAE: 159.98, MAPE: 9.24%) and ETS (MAE: 159.98, MAPE: 9.24%) models with the lowest error rate performed the best. However, the ETS and SES models underpredicted the 2022 forecast compared to reality (1569 and 1572) with reference to the 2020 value, which fell due to the impact of COVID-19. However, working hours, which were 1,877 in 2010, showed a very slow downward trend over the years and remained stable at 1,732 in 2019 and 2021. Therefore, considering that the sharp decline in 2020 was due to COVID-19, the lean method was preferred for the forecast for 2022, taking the same value as the value for 2021 (which was also observed in 2019).

Direct and comprehensive data on the per capita distribution of sports facilities in OECD countries are only available to a limited extent. Therefore, data from official statistical institutions, local sources and map-based prediction models were used together. According to the dataset compiled by Yılmaz, Gündem and Kara (2025), the highest facility density per capita was observed in Sweden (~757/100,000 people), Finland (~600), Iceland (~543) and Canada (~472) [26]. Norway, Switzerland and the Netherlands are also among the countries with high density, while the ratios are lower in Germany, France, Spain, Italy and South Korea. In Türkiye, when only publicly owned facilities are taken into account, this number is 5.2, which is well below the OECD average. For countries where direct data is not available, estimation models were used; for example, it was estimated that the ratios were in the range of 100-200 facilities/100,000 inhabitants for Ireland and 50-150 facilities/100,000 inhabitants for Slovakia. Although the distribution by districts in the UK varies, it is understood that the national average is relatively high.

**Section S3: Model Diagnostics – Linearity, Multicollinearity, and Model Specification**

**Multicollinearity Analysis**

Prior to regression modeling, variance inflation factors (VIF) were calculated to assess the degree of multicollinearity among independent variables in each model. For Model 1 (adolescents), VIF values ranged from 1.29 to 4.12 (highest: internet use, VIF = 4.12). For Model 2 (adults), VIF values were between 1.34 and 3.62 (highest: air pollution DALYs, VIF = 3.62). All VIFs were below the standard threshold of 10, indicating no problematic multicollinearity.

**Model Specification: Ramsey RESET Test**

To verify the correct model specification and functional form, the Ramsey Regression Equation Specification Error Test (RESET) was performed for each model. The test examines whether nonlinear combinations of the fitted values help explain the dependent variable.

- Model 1: RESET = 0.970, df1 = 3, df2 = 26, p = 0.4217
- Model 2: RESET = 0.421, df1 = 3, df2 = 25, p = 0.7395
- Model 3: RESET = 0.857, df1 = 3, df2 = 25, p = 0.4761

All p-values were greater than 0.05, indicating no evidence of model misspecification.

**Linearity and Polynomial Terms**

Linearity between predictors and the logit-transformed outcome was visually checked by plotting LOESS-smoothed curves for each predictor (see Figure S3).

- In Model 1 (adolescents), the addition of quadratic and cubic terms for each predictor did not improve model fit in a statistically significant way; therefore, all predictors in Model 1 were retained in linear form.
- In Model 2 (adults), a significant nonlinear (cubic) relationship was detected for the air pollution-related DALY variable (ages 20+). According to ANOVA test results, the 3rd order polynomial relationship was found to be statistically significant only for the air pollution-related health burden variable for adults (F = 5.0833, p = 0.03071). Since the p-values for all other variables were greater than 0.05, the relationships for those predictors and the logit-transformed adult physical activity rate were considered linear. Initially, the inclusion of quadratic and cubic terms led to very high multicollinearity (standard VIFs: quadratic = 191.29, cubic = 867.15, linear = 293.97). To solve this problem, the orthogonal polynomials approach was applied and the generalized VIF (GVIF) values were recalculated. The GVIF value of the third-order orthogonal polynomial was reduced to 8.23, and the GVIF values of all other independent variables were below 4—well within acceptable limits. This approach ensured that the model could reliably estimate the influence of each polynomial term without instability caused by high multicollinearity.
- In Model 3 (older adults), all variables exhibited linear relationships with the dependent variable, so only linear terms were used.

| **Linearity and LOESS**  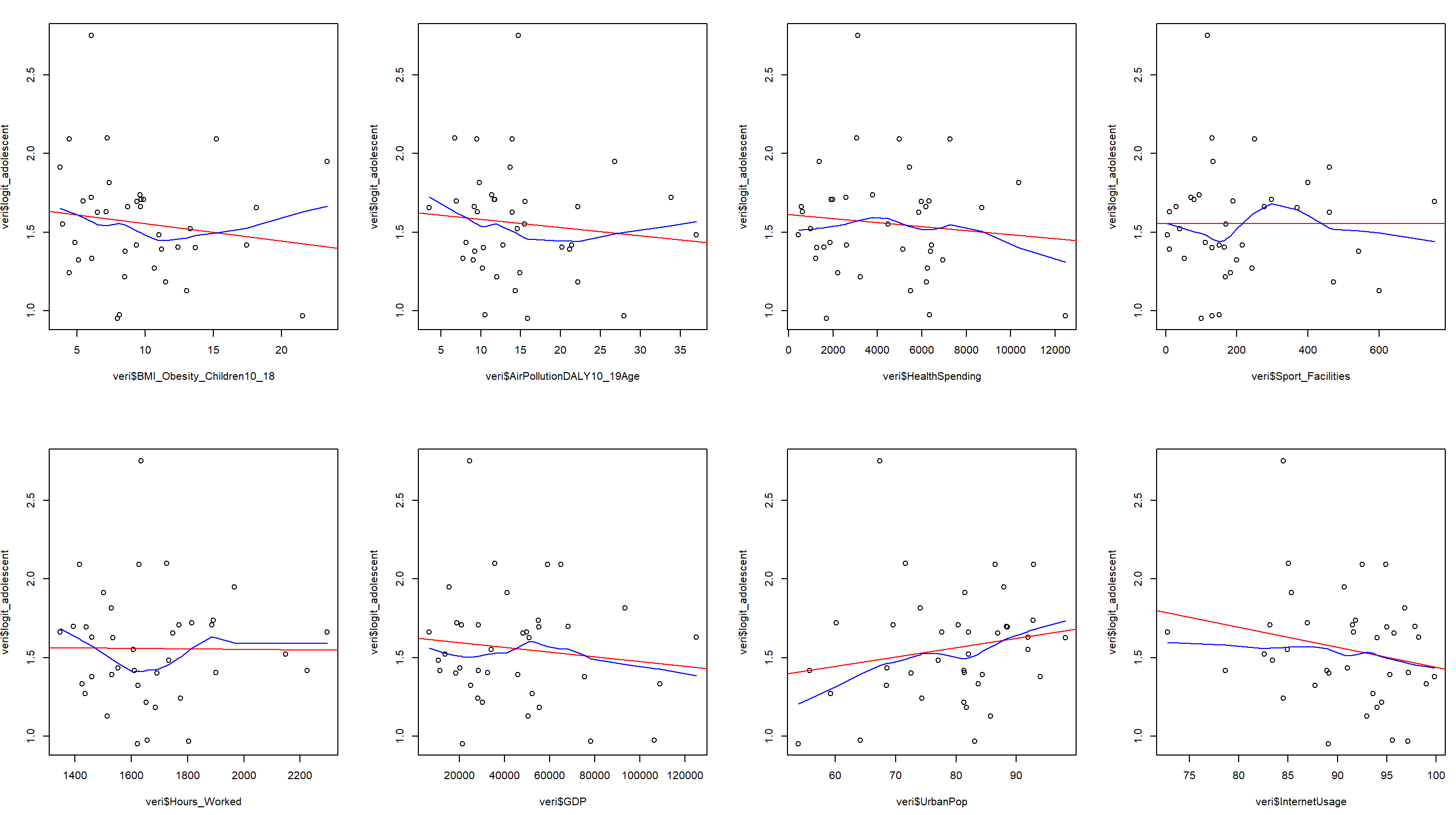  Model 1 |
| --- |
| 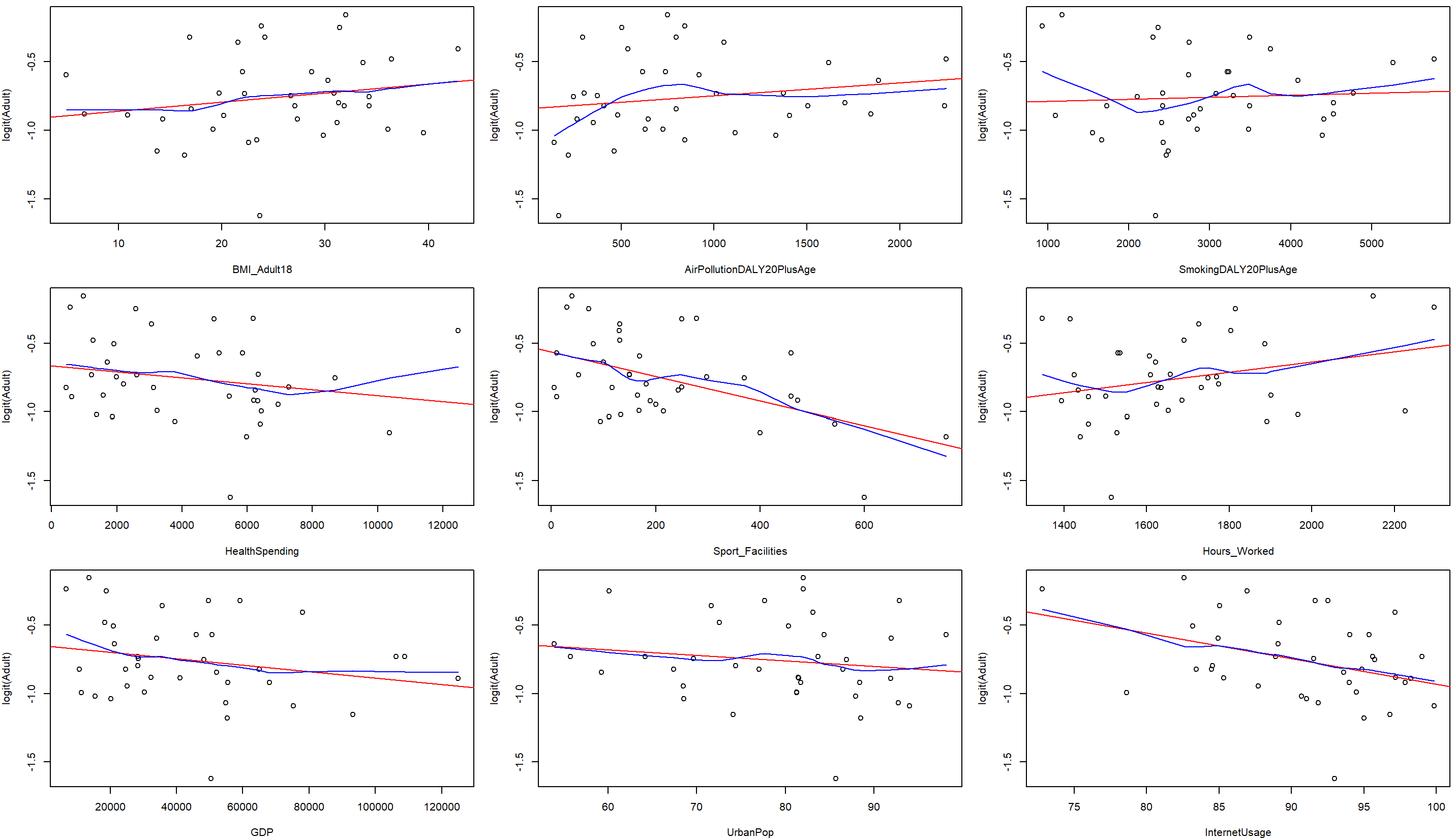  Model 2 |
| 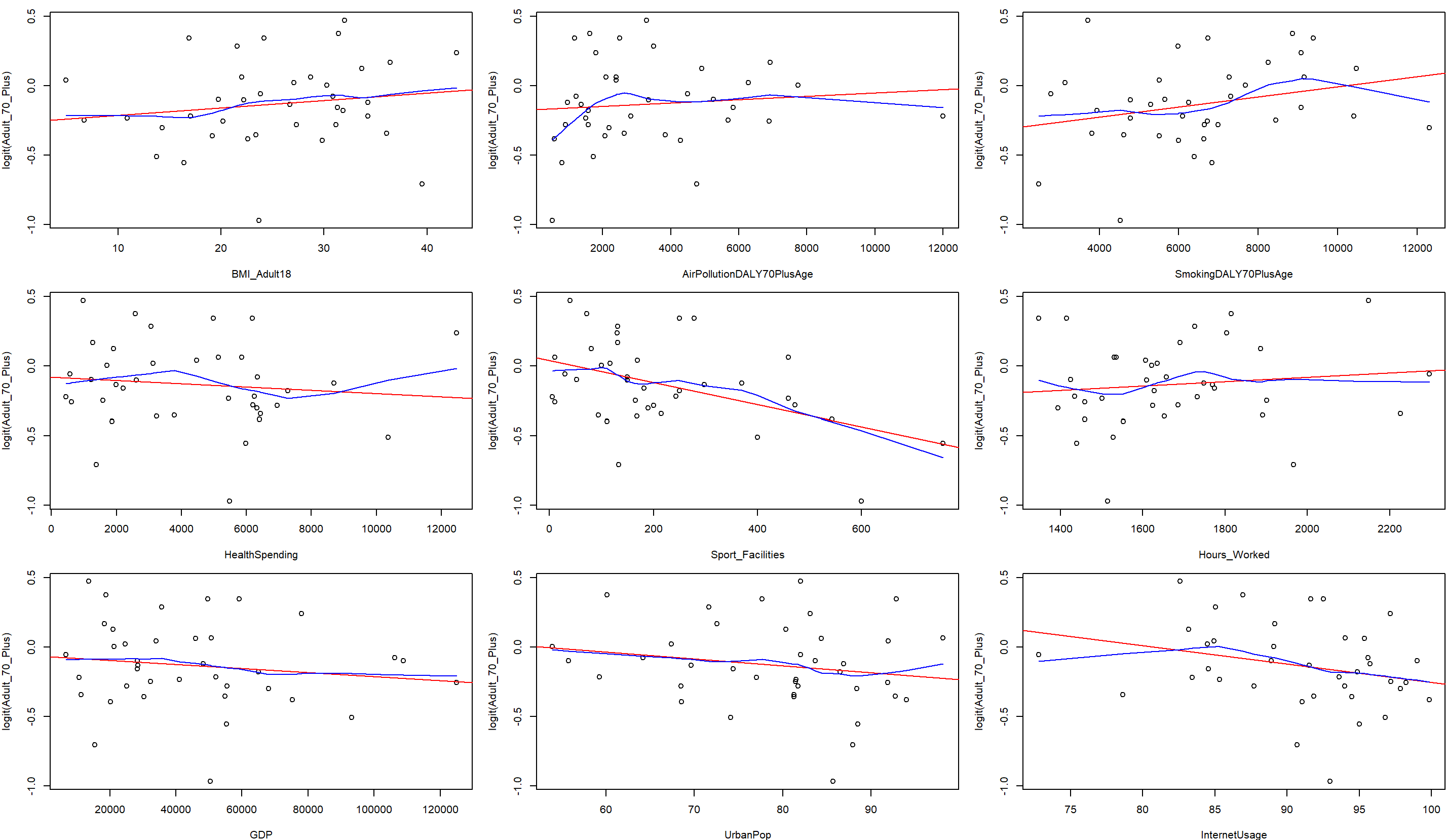  Model 3 |

**Fig S3.** Linearity check before regression model

Overall, model diagnostics confirmed that the relationships between predictors and the logit-transformed outcome were appropriately specified for each age group. In Model 1 (adolescents) and Model 3 (older adults), only linear terms were retained, as adding quadratic or cubic terms did not significantly improve model fit. In Model 2 (adults), however, a significant non-linear association was detected for the air pollution-related DALY variable, warranting the inclusion of a cubic term. Multicollinearity introduced by higher-order terms in Model 2 was successfully addressed using orthogonal polynomials, ensuring all variance inflation factors were within acceptable limits. Diagnostic visualizations using LOESS curves are provided in Figure S3 to support these modeling choices.

**References**

1. World Health Organization. Physical activity. https://www.who.int/news-room/fact-sheets/detail/physical-activity. Accessed March 24, 2025. 2022.

2. World Health Organization. Mortality rate from NCDs. WHO Global Health Observatory. https://www.who.int/data/gho/data/themes/topics/topic-details/GHO/ncd-mortality (Accessed March 31, 2025). 2022.

3. World Bank. GDP per capita (current US$). Retrieved from https://data.worldbank.org/indicator/NY.GDP.PCAP.CD (Accessed September 2, 2025). 2025.

4. World Health Organization. Prevalence of obesity among adults: BMI ≥ 30 (crude estimate) (%). https://www.who.int/data/gho/data/indicators/indicator-details/GHO/prevalence-of-obesity-among-adults-bmi–30-(crude-estimate)-(-) (Accessed March 31, 2025). 2022.

5. World Health Organization. Prevalence of obesity among children and adolescents: BMI > +2 standard deviations above the median (crude estimate) (%). https://www.who.int/data/gho/data/indicators/indicator-details/GHO/prevalence-of-obesity-among-children-and-adolescents-bmi-2-standard-deviations-above-the-median-(crude-estimate)-(-) (Accessed March 31, 2025). 2022.

6. World Bank. Urban population (% of total population). https://data.worldbank.org/indicator/SP.URB.TOTL.IN.ZS (Accessed March 31, 2025). 2022.

7. World Bank. Current health expenditure per capita (current US$). https://data.worldbank.org/indicator/SH.XPD.CHEX.PC.CD (Accessed March 31, 2025). 2025.

8. OECD. Average annual hours actually worked per worker. https://www.oecd.org/en/data/indicators/hours-worked.html?oecdcontrol-d7f68dbeee-var3=2023 (Accessed March 31, 2025). 2022.

9. International Telecommunication Union. Individuals using the Internet (% of population). https://datahub.itu.int/data/?e=1&i=11624 (Accessed March 31, 2025). 2022.

10. Institute for Health Metrics and Evaluation. Global Burden of Disease Study 2021 (GBD 2021) results: Air pollution by age group (10–19, 20+, 70+ years). https://vizhub.healthdata.org/gbd-results/ (Accessed March 31, 2025). 2021.

11. Institute for Health Metrics and Evaluation. Global Burden of Disease Study 2021 (GBD 2021) results: Smoking by age group (10–19, 20+, 70+ years). https://vizhub.healthdata.org/gbd-results/ (Accessed March 31, 2025). 2021.

12. World Bank. Population, male (% of total population). https://data.worldbank.org/indicator/SP.POP.TOTL.MA.ZS?end=2023&name_desc=false&start=1960&view=chart (Accessed March 31, 2025). 2022.

13. World Bank. Population, female (% of total population). https://data.worldbank.org/indicator/SP.POP.TOTL.FE.ZS?end=2023&name_desc=false&start=1960&view=chart (Accessed March 31, 2025). 2022.

14. Rusesji JE, Maresova K. Economic Freedom, Sport Policy, and Individual Participation in Physical Activity: An International Comparison. Contemp Econ Policy. 2014;32:42–55. https://doi.org/10.1111/coep.12027.

15. Yang H, An R, Clarke CV, Shen J. Impact of economic growth on physical activity and sedentary behaviors: a Systematic Review. Public Health. 2023;215:17–26. https://doi.org/10.1016/j.puhe.2022.11.020.

16. Ewing R, Cervero R. Travel and the Built Environment. Journal of the American Planning Association. 2010;76:265–94. https://doi.org/10.1080/01944361003766766.

17. Martin SL, Kirkner GJ, Mayo K, Matthews CE, Durstine; JL, Hebert JR. Urban, Rural, and Regional Variations in Physical Activity. The Journal of Rural Health. 2005;21:239–44. https://doi.org/10.1111/j.1748-0361.2005.tb00089.x.

18. Sallis JF, Floyd MF, Rodríguez DA, Saelens BE. Role of Built Environments in Physical Activity, Obesity, and Cardiovascular Disease. Circulation. 2012;125:729–37. https://doi.org/10.1161/CIRCULATIONAHA.110.969022.

19. Penglee N, Christiana RW, Battista RA, Rosenberg E. Smartphone Use and Physical Activity among College Students in Health Science-Related Majors in the United States and Thailand. Int J Environ Res Public Health. 2019;16:1315. https://doi.org/10.3390/ijerph16081315.

20. Aubert S, Brazo-Sayavera J, González SA, Janssen I, Manyanga T, Oyeyemi AL, et al. Global prevalence of physical activity for children and adolescents; inconsistencies, research gaps, and recommendations: a narrative review. International Journal of Behavioral Nutrition and Physical Activity. 2021;18:81. https://doi.org/10.1186/s12966-021-01155-2.

21. Kaczynski AT, Henderson KA. Environmental Correlates of Physical Activity: A Review of Evidence about Parks and Recreation. Leis Sci. 2007;29:315–54. https://doi.org/10.1080/01490400701394865.

22. Potwarka LR, Kaczynski AT, Flack AL. Places to Play: Association of Park Space and Facilities with Healthy Weight Status among Children. J Community Health. 2008;33:344–50. https://doi.org/10.1007/s10900-008-9104-x.

23. McCormack GR, Shiell A. In search of causality: a systematic review of the relationship between the built environment and physical activity among adults. International Journal of Behavioral Nutrition and Physical Activity. 2011;8:125. https://doi.org/10.1186/1479-5868-8-125.

24. Powell LM, Slater S, Chaloupka FJ, Harper D. Availability of Physical Activity–Related Facilities and Neighborhood Demographic and Socioeconomic Characteristics: A National Study. Am J Public Health. 2006;96:1676–80. https://doi.org/10.2105/AJPH.2005.065573.

25. Lee SA, Ju YJ, Lee JE, Hyun IS, Nam JY, Han K-T, et al. The relationship between sports facility accessibility and physical activity among Korean adults. BMC Public Health. 2016;16:893. https://doi.org/10.1186/s12889-016-3574-z.

26. Yılmaz S, Gündem S, Kara SB. Access to sport facilities in OECD countries (per 100,000 population), 2022 [Data set]. Zenodo. https://doi.org/10.5281/zenodo.15114349 . 2025.

27. Sahoo K, Sahoo B, Choudhury A, Sofi N, Kumar R, Bhadoria A. Childhood obesity: causes and consequences. J Family Med Prim Care. 2015;4:187. https://doi.org/10.4103/2249-4863.154628.

28. Saunders TJ, Gray CE, Poitras VJ, Chaput J-P, Janssen I, Katzmarzyk PT, et al. Combinations of physical activity, sedentary behaviour and sleep: relationships with health indicators in school-aged children and youth. Applied Physiology, Nutrition, and Metabolism. 2016;41 6 (Suppl. 3):S283–93. https://doi.org/10.1139/apnm-2015-0626.

29. An R, Zhang S, Ji M, Guan C. Impact of ambient air pollution on physical activity among adults: a systematic review and meta-analysis. Perspect Public Health. 2018;138:111–21. https://doi.org/10.1177/1757913917726567.

30. Shields KN, Cavallari JM, Hunt MJO, Lazo M, Molina M, Molina L, et al. Traffic-related air pollution exposures and changes in heart rate variability in Mexico City: A panel study. Environmental Health. 2013;12:7. https://doi.org/10.1186/1476-069X-12-7.

31. Han B, Zhao R, Zhang N, Xu J, Zhang L, Yang W, et al. Acute cardiovascular effects of traffic-related air pollution (TRAP) exposure in healthy adults: A randomized, blinded, crossover intervention study. Environmental Pollution. 2021;288:117583. https://doi.org/10.1016/j.envpol.2021.117583.

32. D’Oliveira A, Dominski FH, De Souza LC, Branco JHL, Matte DL, da Cruz WM, et al. Impact of air pollution on the health of the older adults during physical activity and sedentary behavior: A systematic review. Environ Res. 2023;234:116519. https://doi.org/10.1016/j.envres.2023.116519.

33. Tainio M, de Nazelle AJ, Götschi T, Kahlmeier S, Rojas-Rueda D, Nieuwenhuijsen MJ, et al. Can air pollution negate the health benefits of cycling and walking? Prev Med (Baltim). 2016;87:233–6. https://doi.org/10.1016/j.ypmed.2016.02.002.

34. Xu J, Zhou J, Luo P, Mao D, Xu W, Nima Q, et al. Associations of long-term exposure to ambient air pollution and physical activity with insomnia in Chinese adults. Science of The Total Environment. 2021;792:148197. https://doi.org/10.1016/j.scitotenv.2021.148197.

35. Ng R, Sutradhar R, Yao Z, Wodchis WP, Rosella LC. Smoking, drinking, diet and physical activity—modifiable lifestyle risk factors and their associations with age to first chronic disease. Int J Epidemiol. 2020;49:113–30. https://doi.org/10.1093/ije/dyz078.

36. Katzmarzyk PT, Friedenreich C, Shiroma EJ, Lee I-M. Physical inactivity and non-communicable disease burden in low-income, middle-income and high-income countries. Br J Sports Med. 2022;56:101–6. https://doi.org/10.1136/bjsports-2020-103640.

37. Sato M, Du J, Inoue Y, Funk DC, Weaver F. Older Adults’ Physical Activity and Healthcare Costs, 2003–2014. Am J Prev Med. 2020;58:e141–8. https://doi.org/10.1016/j.amepre.2019.12.009.

38. Carlson SA, Fulton JE, Pratt M, Yang Z, Adams EK. Inadequate Physical Activity and Health Care Expenditures in the United States. Prog Cardiovasc Dis. 2015;57:315–23. https://doi.org/10.1016/j.pcad.2014.08.002.

39. Foster C, Hillsdon M. Changing the environment to promote health-enhancing physical activity. J Sports Sci. 2004;22:755–69. https://doi.org/10.1080/02640410410001712458.

40. Laddu D, Paluch AE, LaMonte MJ. The role of the built environment in promoting movement and physical activity across the lifespan: Implications for public health. Prog Cardiovasc Dis. 2021;64:33–40. https://doi.org/10.1016/j.pcad.2020.12.009.

41. Dishman RK. Exercise, fitness, and health: A consensus of current knowledge. In: Bouchard C, Shephard RJ, Stephens T, Sutton JR, McPherson BD, editors. Determinants of participation in physical activity. 1st edition. Human Kinetics; 1990. p. 75–102.

42. Dishman RK, Sallis JF, Orenstein DR. The determinants of physical activity and exercise. Public Health Reports. 1985;100:158–71.

43. Angrave D, Charlwood A, Wooden M. Long working hours and physical activity. J Epidemiol Community Health (1978). 2015;69:738–44. https://doi.org/10.1136/jech-2014-205230.

44. World Health Organization. Global status report on physical activity 2022. Available from: https://www.who.int/publications/i/item/9789240059153 [cited 2025 July 21]. Geneva; 2022.

45. Guthold R, Stevens GA, Riley LM, Bull FC. Worldwide trends in insufficient physical activity from 2001 to 2016: a pooled analysis of 358 population-based surveys with 1·9 million participants. Lancet Glob Health. 2018;6:e1077–86. https://doi.org/10.1016/S2214-109X(18)30357-7.

46. Lee I-M, Shiroma EJ, Lobelo F, Puska P, Blair SN, Katzmarzyk PT. Effect of physical inactivity on major non-communicable diseases worldwide: an analysis of burden of disease and life expectancy. The Lancet. 2012;380:219–29. https://doi.org/10.1016/S0140-6736(12)61031-9.

47. White IR, Royston P, Wood AM. Multiple imputation using chained equations: Issues and guidance for practice. Stat Med. 2011;30:377–99. https://doi.org/10.1002/sim.4067.

48. Hacker K. The Burden of Chronic Disease. Mayo Clin Proc Innov Qual Outcomes. 2024;8:112–9. https://doi.org/10.1016/j.mayocpiqo.2023.08.005.

49. Booth FW, Roberts CK, Laye MJ. Lack of Exercise Is a Major Cause of Chronic Diseases. In: Comprehensive Physiology. Wiley; 2012. p. 1143–211. https://doi.org/10.1002/cphy.c110025.

50. Carlson SA, Adams EK, Yang Z, Fulton JE. Percentage of Deaths Associated With Inadequate Physical Activity in the United States. Prev Chronic Dis. 2018;15:170354. https://doi.org/10.5888/pcd18.170354.

51. Xu F, Weng Y, Lv D, Zheng H, Xiong J, Zhang L, et al. Applied theory of planned behavior to explore associated factor of physical activity level among Chinese childhood cancer survivors. Journal of Cancer Survivorship. 2025. https://doi.org/10.1007/s11764-025-01768-7.
